# Increased methylation and decreased expression of homeobox genes *TLX1*, *HOXA10* and *DLX5* in human placenta are associated with trophoblast differentiation

**DOI:** 10.1038/s41598-017-04776-5

**Published:** 2017-07-03

**Authors:** Boris Novakovic, Thierry Fournier, Lynda K. Harris, Joanna James, Claire T. Roberts, Hannah E. J. Yong, Bill Kalionis, Danièle Evain-Brion, Peter R. Ebeling, Euan M. Wallace, Richard Saffery, Padma Murthi

**Affiliations:** 1 Cancer and Disease Epigenetics, Murdoch Childrens Research Institute, Royal Children’s Hospital, Victoria, Australia; 2 INSERM, UMR-S1139, Faculté des Sciences Pharmaceutiques et Biologiques, Paris, France; 3 Université Paris Descartes, Paris, France; 4 Fondation PremUp, Paris, France; 5 Manchester Pharmacy School, The University of Manchester, Manchester, UK; 6 Department of Obstetrics and Gynaecology, University of Auckland, Auckland, New Zealand; 7 Robinson Research Institute, and Adelaide Medical School, University of Adelaide, North Adelaide, South Australia Australia; 8 Department of Maternal-Fetal Medicine Pregnancy Research Centre, The Royal Women’s Hospital, and Department of Obstetrics and Gynaecology, University of Melbourne, Victoria, Australia; 9 Department of Medicine, School of Clinical Sciences, Monash University, Victoria, Australia; 10 The Ritchie Centre, Hudson Institute of Medical Research, Victoria, Australia; 11 Department of Obstetrics and Gynaecology, Monash University, Victoria, Australia; 12 Department of Paediatrics, University of Melbourne, Victoria, Australia

## Abstract

Homeobox genes regulate embryonic and placental development, and are widely expressed in the human placenta, but their regulatory control by DNA methylation is unclear. DNA methylation analysis was performed on human placentae from first, second and third trimesters to determine methylation patterns of homeobox gene promoters across gestation. Most homeobox genes were hypo-methylated throughout gestation, suggesting that DNA methylation is not the primary mechanism involved in regulating HOX genes expression in the placenta. Nevertheless, several genes showed variable methylation patterns across gestation, with a general trend towards an increase in methylation over gestation. Three genes (*TLX1, HOXA10* and *DLX5*) showed inverse gains of methylation with decreasing mRNA expression throughout pregnancy, supporting a role for DNA methylation in their regulation. Proteins encoded by these genes were primarily localised to the syncytiotrophoblast layer, and showed decreased expression later in gestation. siRNA mediated downregulation of *DLX5*, *TLX1* and *HOXA10* in primary term villous cytotrophoblast resulted in decreased proliferation and increased expression of differentiation markers, including *ERVW-1*. Our data suggest that loss of *DLX5, TLX1* and *HOXA10* expression in late gestation is required for proper placental differentiation and function.

## Introduction

The human placenta provides a critical interface between the maternal and fetal circulations during pregnancy. Formation of this interface is controlled by growth factors, cytokines and nuclear transcription factors. Cells respond to cues from growth factors and signalling molecules by either maintaining or altering their state of differentiation. Within cells, transcription factors play essential roles in regulating cell proliferation and differentiation and this is evident in the most important placental lineage; trophoblast cells^1–4^. The numerous cellular transcription factors that control proliferation and differentiation fall into a few families based on the presence of conserved motifs, which include the zinc finger, leucine zipper, helix-loop-helix, helix-turn-helix and homeobox domains^5, 6^.

The homeobox gene family plays fundamental roles in embryonic pattern formation and morphogenesis^7–9^. These genes were identified in mammals and vertebrates based on sequence homology to the genes of the *Drosophila* HOM-C complex^8, 10, 11^. In humans, the homeobox complex comprises 39 genes arranged in four separate chromosomal clusters; *HOX A, B, C* and *D* ^12–14^ – together into various subfamilies based on their functional and structural characteristics, and these subfamilies are essential for the control of specific aspects of cellular growth and differentiation^14^.

Homeobox genes play major roles in controlling extraembryonic development of the placenta. Studies of homeobox genes in the human placenta initially focused on identifying homeobox genes expressed in the normal placenta and in trophoblastic cancers^9, 15, 16^. Subsequently, we and others identified human homeobox genes *DLX3*, *DLX4*, *GAX*, *ESX1L* and *HLX*, which play crucial roles in embryogenesis and organogenesis, as potentially important in normal and pathological human placental development^17–26^. We showed *HLX* expression is primarily in proliferating cytotrophoblast cell types and in mesenchymal stem/stromal cells during placental development^25, 27^ and that *HLX* expression decreases in human idiopathic fetal growth restriction^20^. In cell culture models, we showed that *HLX* regulates trophoblast cell migration^28^. Targets of *HLX* included genes involved in cell cycle regulation and other aspects of proliferation^29^.

Homeobox gene *DLX4* functional studies revealed this gene was a regulator of trophoblast cell apoptosis and had a potential role in epithelial-mesenchymal transition^26, 30^. With regard to homeobox gene *DLX3*, we showed its expression was localised to nuclei of proliferating villous and extravillous cytotrophoblast cells, as well as in nuclei within the syncytiotrophoblast layer in the human placenta^17^. *DLX3* target genes are associated with trophoblast differentiation, including *GATA2* and *PPARG* that encode GATA binding protein 2 and peroxisome proliferator-activated receptor γ respectively^31^, *CGB*, the beta subunit of hCG^18, 32^, and 3-βHSD^18, 32^. Finally, we and others have identified likely direct or indirect target genes downstream for homeobox genes that are involved in the regulation of endothelial functions^33^.

Whilst studies are progressing on growth factor regulation of homeobox genes and target gene identification in normal and pathological human placental development, our understanding of how external or environmental factors regulate homeobox gene expression in extraembryonic tissues, most likely via epigenetic mechanisms, is manifestly inadequate. We previously showed that DNA methylation (the most widely studied epigenetic process) is dynamic during placental development, with genome-scale changes in methylation patterns between first and third trimester placenta^34^. Many studies have now investigated the association between DNA methylation and placental function, including the potential role as a mediator of placental dysfunction in adverse pregnancy outcomes of preeclampsia, gestational diabetes and intra uterine growth restriction^35^. Thus epigenetic modifications can regulate families of genes in the placenta during gestation. DNA methylation is one mechanism by which HOX gene expression is modulated, with several HOX gene loci showing tissue-specific DNA methylation patterns^36, 37^. Indeed, the HOX family of genes are more likely to show tissue-specific DNA methylation profiles compared with non-developmentally essential genes^38^.

In this study, we took a gene family-specific approach to determine the role of DNA methylation in regulating homeobox genes during placental development. Specifically, we identified homeobox genes showing an inverse relationship between DNA methylation and gene expression status from first to third trimester. Such genes are candidates for epigenetic regulation during placental development. Determining the temporal, epigenetic control of homeobox gene expression during placental development could provide insights into the function of these genes in placentation and in adverse pregnancy outcomes.

## Results

### Identification of differentially methylated homeobox family genes in the placenta across gestation

**Figure 1:**
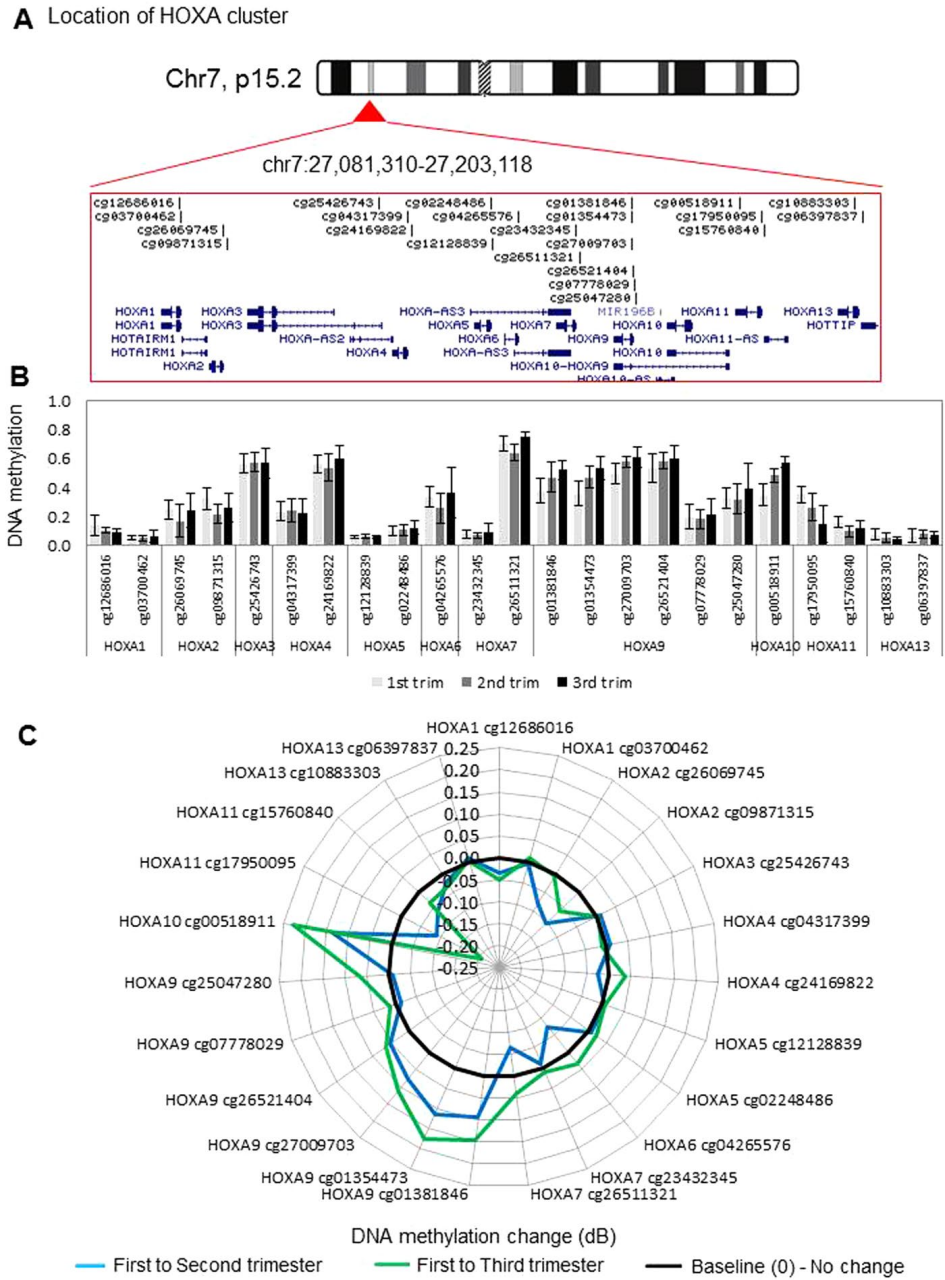
DNA methylation levels and dynamics at the HOXA gene cluster. (**A**) Location and organization of the HOXA gene cluster on chromosome 7, with the location of genes and HM27 probes shown. (**B**) Bar plot showing mean DNA methylation level (beta-value) for each probe located within the HOXA locus, sorted by gene. For first, second and third trimester, n = 18, 10, and 14 were used, respectively. Error bars are standard deviation. (**C**) Radar plot showing change in methylation (delta-Beta) for each probe within the HOXA cluster in second and third trimester, relative to first trimester placenta. Black line marks 0 (no change in methylation), positive values indicate increase in methylation and negative values indicate decrease in methylation over gestation.

**Figure 2:**
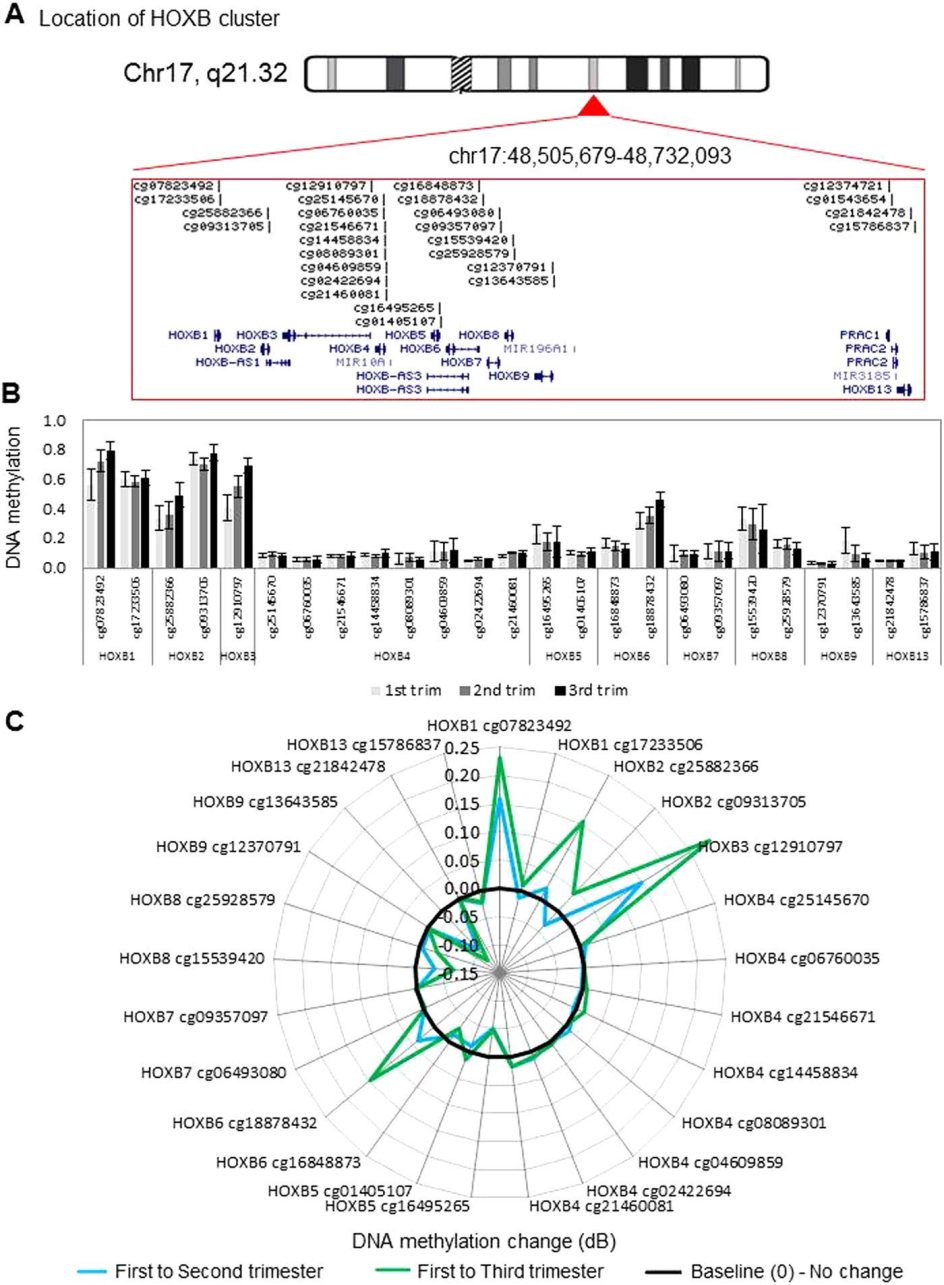
DNA methylation levels and dynamics at the HOXB gene cluster. (**A**) Location and organization of the HOXB gene cluster on chromosome 17, with the location of genes and HM27 probes shown. (**B**) As in Fig. 1 legend above but for HOXB locus, sorted by gene. For first, second and third trimester, n = 18, 10, and 14 were used, respectively. Error bars are standard deviation. (**C**) As in Fig. 1 legend above but for each probe within the HOXB cluster in second and third trimester, relative to first trimester placenta. Black line marks 0 (no change in methylation), positive values indicate increase in methylation and negative values indicate decrease in methylation over gestation.

**Figure 3:**
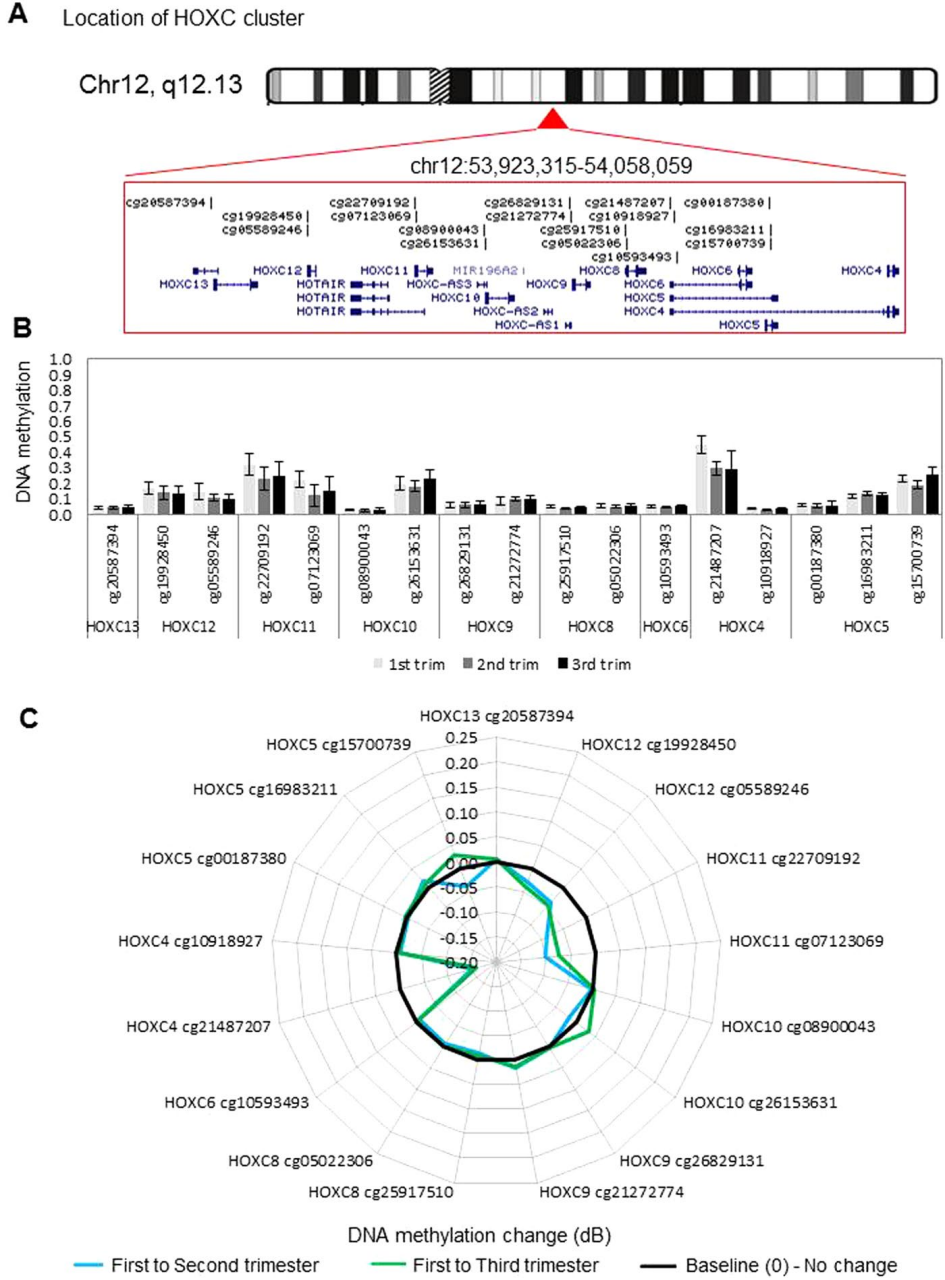
DNA methylation levels and dynamics at the HOXC gene cluster. (**A**) Location and organization of the HOXC gene cluster on chromosome 12, with the location of genes and HM27 probes shown. (**B**) As in Fig. 1 legend above but for HOXC locus, sorted by gene. (**C**) As in Fig. 1 legend above but for HOXC cluster. Black line marks 0 (no change in methylation), positive values indicate increase in methylation and negative values indicate decrease in methylation over gestation.

**Figure 4:**
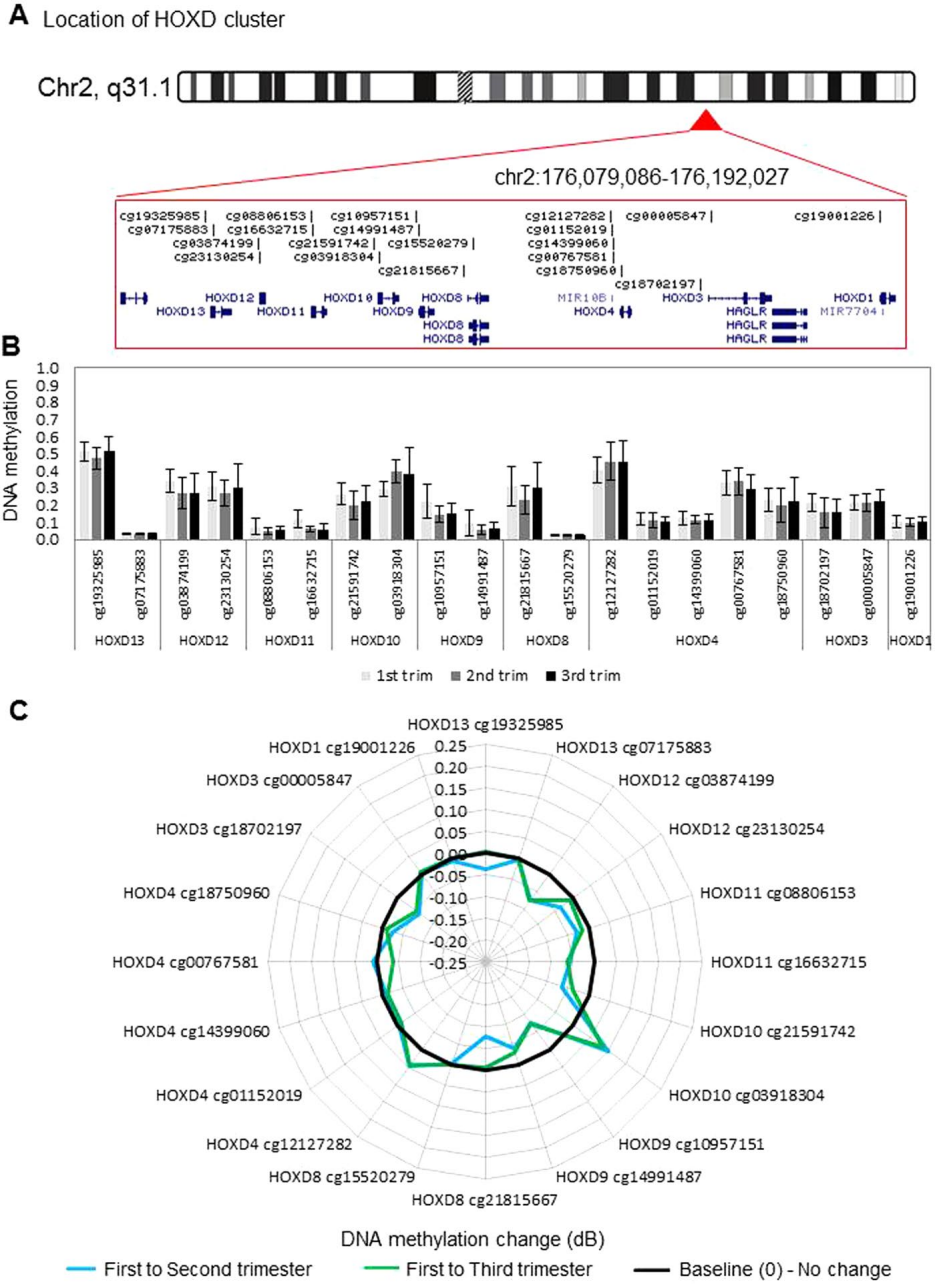
DNA methylation levels and dynamics at the HOXD gene cluster. (**A**) Location and organization of the HOXD gene cluster on chromosome 2, with the location of genes and HM27 probes shown. (**B**) As in Fig. 1 legend above but for HOXD locus, sorted by gene. (**C**) As in Fig. 1 legend but for each probe within the HOXD cluster in second and third trimester, relative to first trimester placenta. Black line marks 0 (no change in methylation), positive values indicate increase in methylation and negative values indicate decrease in methylation over gestation.

The 35 HOXA-D family genes are located in four clusters on chromosomes 2, 7, 12 and 17, and are covered by 85 probes on the Infinium 27 K methylation array. Additionally we were interested in other genes containing the homeo-box, based on previous associations with placental function or fetal development. The output of the HM27 array consists of a beta value with a range from 0–1, representing 0–100% methylation. Each probe (of the roughly 27,000) on the HM27 array, targets an individual CpG site in the genome, with the array designed in such a way that on average, each gene is targeted by 2 probes (with a range between 1 to >20 probes for certain genes). Using previously published methylation data^34^ (GEO31781) in 18 first, 10 second and 14 third trimester placentae we observe that for the most part HOX genes do not show change in methylation during gestation (Figs 1 to 4). HOXA and HOXB clusters contained several differential CpG sites (Figs 1 and 2), while HOXC and D clusters did not contain any (Fig. 3 and 4). Furthermore, we extended our analysis to profile HOX genes outside the HOXA-D families, and found that the majority of homeobox gene promoters show low methylation at first trimester, and remain hypo-methylated throughout gestation (Figure S1). Homeobox genes for which expression is potentially regulated by promoter DNA methylation were identified as those that showed a change in methylation (delta beta (Δβ)  > 0.1; p < 0.05) from first trimester to term. Two genes, *HOXB9* and *MSX1* (represented by 3 probes), showed a significant loss of methylation (Figures S1 and S2). In contrast, five genes (*DLX5*, *TLX1*, *HOXA10*, *TGIF* and *HOXB6*) showed increases in methylation over gestation (Figure S1). The most compelling finding was *DLX5*, which was represented by 5 probes that all showed the same trend towards increasing methylation with gestational age (Table S1).

### *DLX5* gene body methylation is associated with gene repression

**Figure 5:**
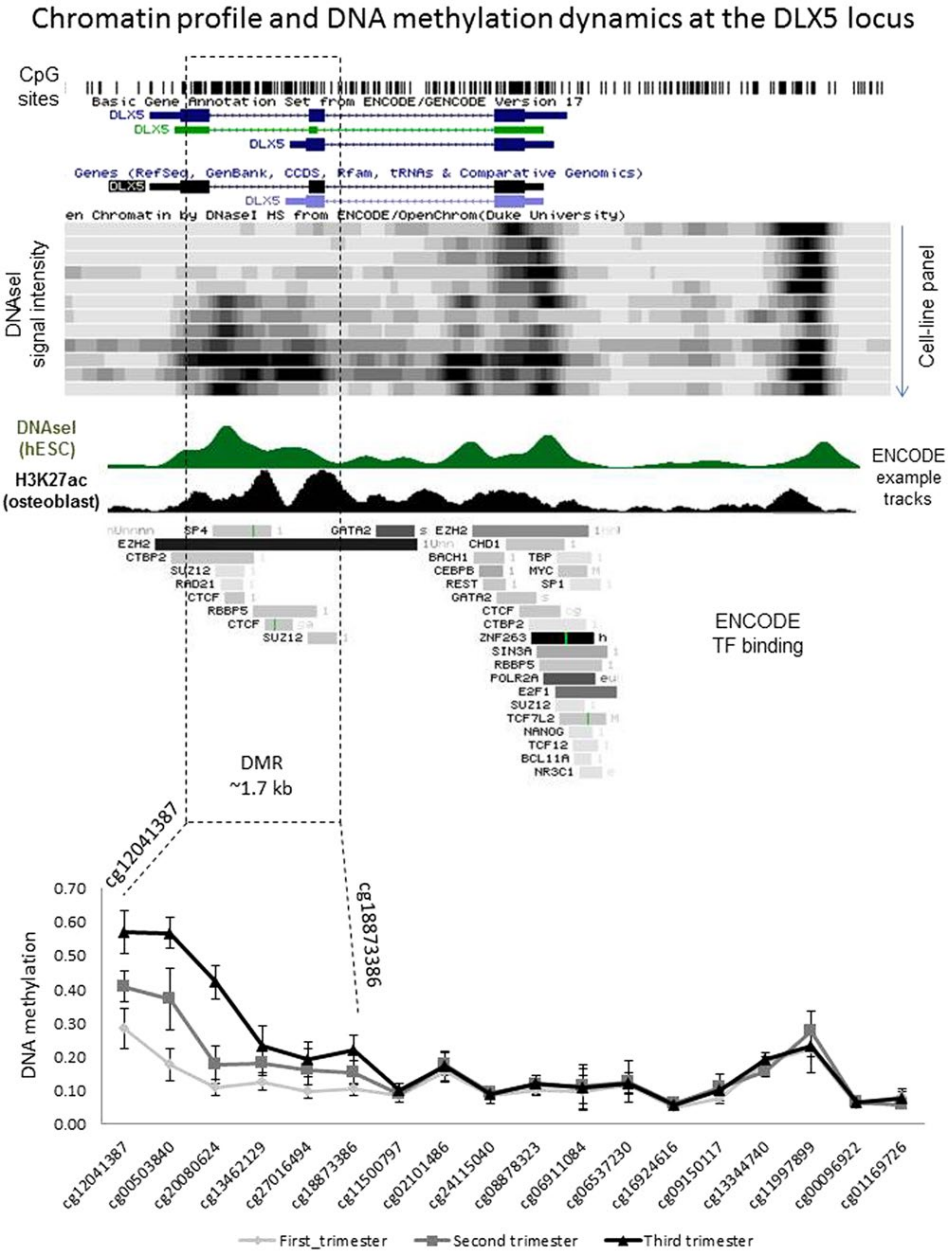
*DLX5* gene body methylation is restricted to the 3′ end *DLX5* had the highest number of associated dynamic CpG sites. By plotting DNA methylation in first, second and third trimester for each site at this gene, it is clear that DNA methylation change is occurring at the 3′ end of the gene, and not at the promoter region. ENCODE DNAseI track shows that the 3′ DMR is associated with open chromatin in a subset of human tissues, indicating that change in DNA methylation may result in change of accessibility.

The *DLX5* gene was covered by 18 probes on the array, of which 6 probes showed significant increases in methylation from the first to third trimester (Fig. 5). The sites of differential methylation were spread over exon 2 and 3 of *DLX5*, and stretch across a region of about 1.7 kb. In order to determine the accessibility of this region, we looked at publically available ENCODE DNAseI tracks on the UCSC genome browser (Fig. 5). Representative DNAseI intensities are shown for several cell lines and primary cells. As expected, all tracks show open chromatin at the promoter region, and several show DNAseI signal within the identified differentially methylated region (DMR). This comparison indicates that DNA methylation change is occurring at a possible regulatory region.

### Relationship between homeobox gene methylation and expression in placenta tissue throughout pregnancy

**Figure 6:**
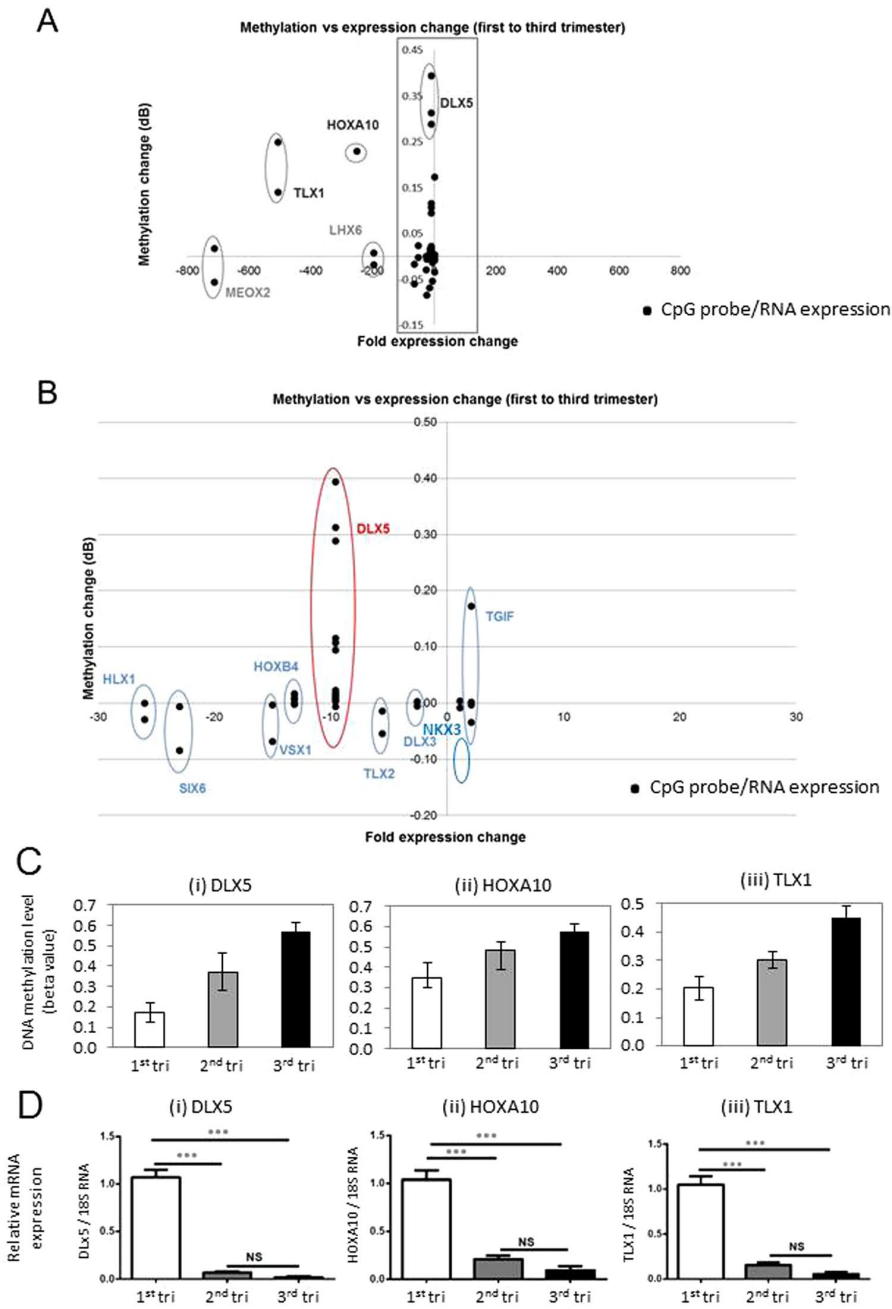
Effect of DNA methylation change at homeobox gene loci on gene expression. (**A**) The correlation between DNA methylation change between first and third trimester (y-axis) and RNA expression change between first and third trimester (x-axis) for each homeobox gene. Eleven out of 13 genes showed lower expression in third trimester compared to first (clustering left of centre). Only 3 genes - DLX5, *HOXA10* and *TLX1* showed a concomitant increase in methylation (clustering in top left corner). (**B**) Shows the magnified view of the boxed area in panel A. DNA methylation data points represent n = 18 first trimester and n = 10 third trimester samples, while gene expression data points represent n = 5 first trimester and n = 2 third trimester samples. (**C**) Change in DNA methylation in second and third trimester compared to first trimester for *DLX5* (HM27 probe cg00503840), *HOXA10* (HM27 probe cg00518911), *TLX1* (HM27 probe cg11498156). The second trimester shows an intermediate level of methylation change compared to third trimester, which indicates that DNA methylation is changing progressively over gestation. The change in methylation at second or third trimester is based on the difference between the mean methylation of these time-points relative to the first trimester time-point (delta Beta). (**D**) Quantitative RT-PCR confirms the reduction in TLX1, HOXA10 and DLX5 expression during gestation. N = 12 first trimester, n = 4 second trimester, and n = 6 third trimester, data are presented as relative expression change (mean+/− SD).

DNA methylation changes (Δβ) from first to third trimester were plotted against fold expression change during the same gestation period. Expression changes were measured for 13 genes using real-time PCR. Overall, increasing gestation was associated with a loss of homeobox gene expression, with 11 out of 13 homeobox genes showing lower expression in the third trimester (Fig. 6). For most genes, loss of expression was not associated with an increase in DNA methylation (Fig. 6A and B), suggesting that DNA methylation is not the primary mechanism of regulating expression of these genes. Only three genes (*TLX1, HOXA10* and *DLX5*) showed a reciprocal gain of methylation with decreasing expression over time (Fig. 6A) suggesting a role for DNA methylation in gene regulation. Interestingly, for each of *TLX1, HOXA10* and *DLX5*, methylation increased progressively over gestation, with second trimester showing an intermediate level of methylation to that of first and third trimester villi (Fig. 6C). In order to confirm that the DNA methylation changes observed at the HM27 CpG probes were representative of the surrounding region, we used published HM450 array data on 5 first and 10 third trimester placentas, from an unrelated cohort (GSE49343)^39^. We show that the *HOXA10* differential probe is in a DMR with 5 CpG sites showing increased methylation over gestation (Figure S3). The *DLX5* probes are in a large DMR containing 12 differential CpG probes (Figure S4) and the *TLX1* probes are in a region containing 4 differential CpG probes (Figure S5). The change in expression of *DLX5*, *TLX1* and *HOXA10* across gestation was investigated by quantitative RT-PCR on first, second and third trimester placental villi (Fig. 6D). These analyses showed a significant decrease in expression of all three genes in second trimester and third trimester placentae compared with first trimester placental tissues (Fig. 6D).

### DLX5, HOXA10 and TLX1 are localised in the nuclei of the syncytiotrophoblast of the first-trimester and term placentae

**Figure 7:**
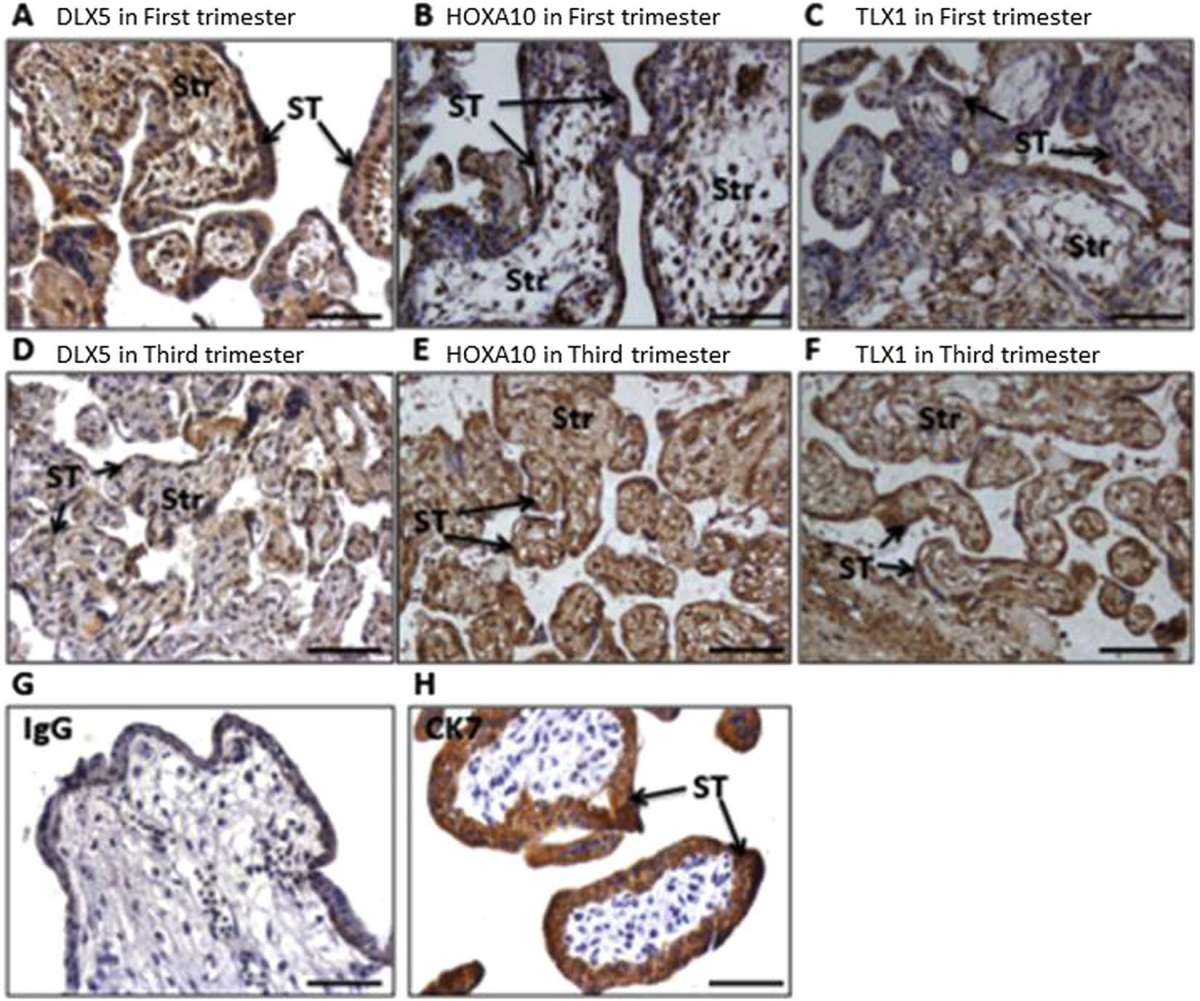
Localisation of DLX5, HOXA10 and TLX1 proteins in first and third trimester placentae Representative sections of n = 5 first trimester and n = 5 third trimester placentae. DLX5, HOXA10 and TLX1 are expressed in the syncytiotrophoblast layer (black arrows) of first trimester (**A–C**) and third trimester (**D–F**) placentae. CK7 was used as a positive marker (**H**) and IgG as a negative control (**G**). (**I**) Dot plot shows immunoreactive signal pixel density for DLX5, HOXA10 and TLX1 in first and third trimester villi. Intensity for all three candidate proteins was significantly lower in the third trimester. The intensity of staining was calculated using an AxioVision software (Carl Zeiss KS 400, Germany) from five fields of view of each placental section and the results were expressed as the mean pixel density (in arbitrary units). Str = stromal.

The DNA methylation and gene expression data suggest a progressive silencing of *DLX5*, *TLX1* and *HOXA10* expression in placental villi during gestation. We therefore sought to determine the specific cell compartments to which these proteins are localised. Figure 7A demonstrates that DLX5, HOXA10 and TLX1 protein was localised primarily to the nuclei of syncytiotrophoblast in both first-trimester (Panel A–C) and term placental tissues (Panel D–F). Weaker expression of these genes was also detected in the villous stroma. Non-immune rabbit IgG was used as a negative control (Panel G) and CK7 was used as a positive control (Panel H). Figure 7I shows the quantitative analysis of the staining intensity for DLX5, HOXA10 and TLX1. The staining intensity was significantly decreased in term placental tissues (n = 5) for all three candidate proteins compared with first-trimester placentae (n = 5).

### Functional role of *DLX5*, *HOXA10* and *TLX1* in primary term trophoblasts

**Figure 8:**
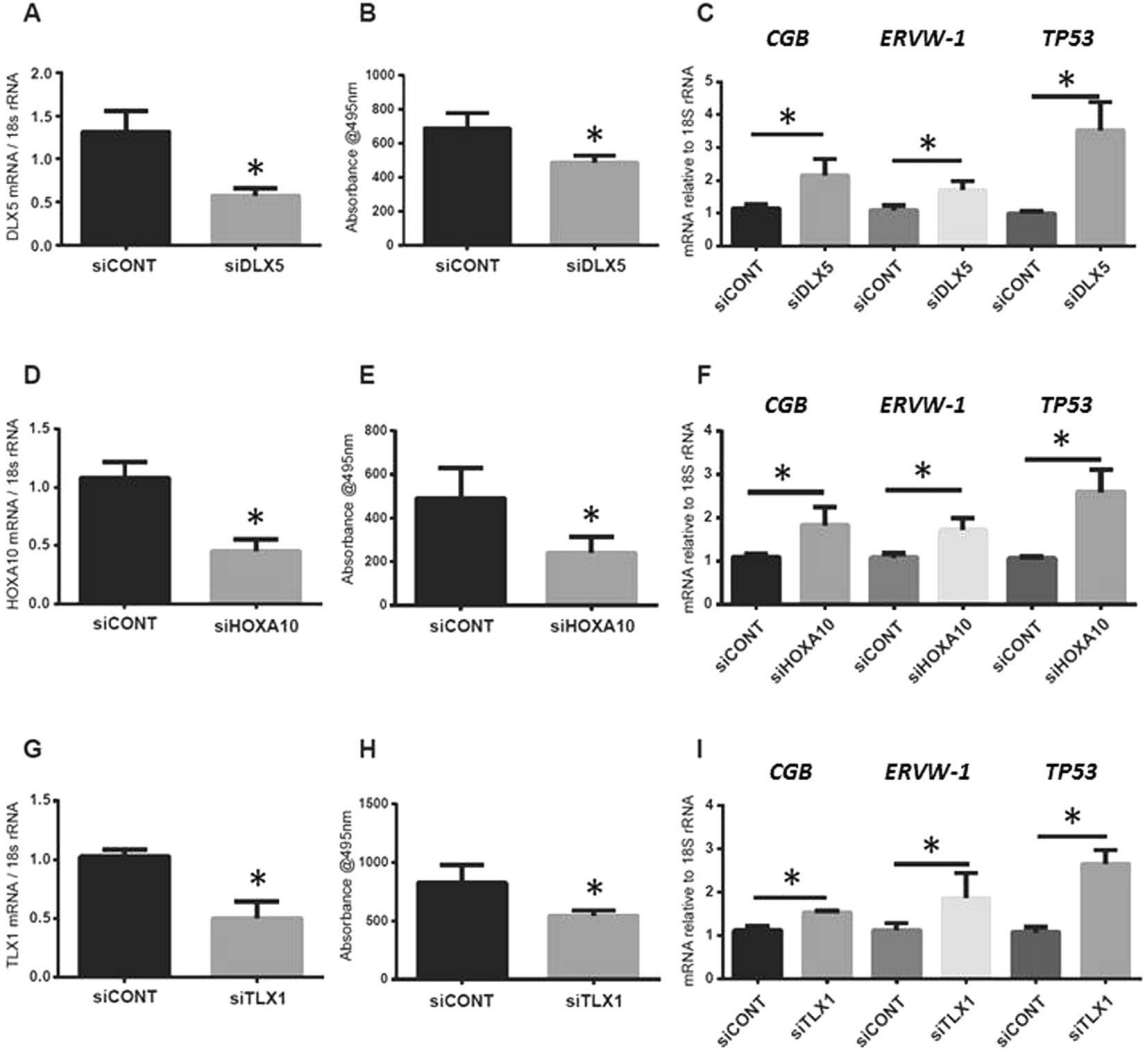
Reduction of DLX5, HOXA10 and TLX1 expression in trophoblasts using siRNA leads to lower proliferation and higher expression of differentiation markers siRNA specific for DLX5, HOXA10 and TLX1 were used to knock-down their expression in primary trophoblasts. Expression of DLX5 (**A**), HOXA10 (**D**) and TLX1 (**G**) were reduced by the siRNA. In each case, trophoblast proliferation was reduced (panels B, E, and H). Reduction in DLX5, HOXA10 or TLX1, resulted in increased expression of trophoblast differentiation markers - *CGB*, *ERVW-1* and *TP53* (panels C, F, and I). For panels B, E and H, @495 nm = absorbance at 495 nm.

Figure 8 demonstrates the functional role of *DLX5*, *HOXA10* and *TLX1* in trophoblast proliferation, differentiation and apoptosis following treatment of cultured trophoblasts with specific siRNAs. Gene specific siRNA treatment of trophoblasts significantly decreased *DLX5* (Fig. 8A), *HOXA10* (Fig. 8D) and *TLX1* (Fig. 8G) relative to *18S rRNA* compared with siCONT (scrambled control) treated cells (n = 4 independent experiments, p < 0.05). Functional analyses of trophoblasts demonstrated a reduction in cellular proliferation following treatment with *siDLX5* (Fig. 8B), or *siHOXA10* (Fig. 8E), or *siTLX1* (Fig. 8H) compared with siCONT treated trophoblasts (n = 4 independent experiments, p < 0.05). Furthermore, *CGB*, *ERW-1 (Syncytin-1)* and *TP53* mRNA were also significantly increased following treatment with *siDLX5* (Fig. 8C), or *siHOXA10* (Fig. 8F), or *siTLX1* (Fig. 8I) compared with siCONT treated trophoblasts suggesting that markers of trophoblast differentiation and apoptosis are direct or indirect targets of *DLX5*, *HOXA10* and *TLX1*.

## Discussion

In this study, we report differential methylation of homeobox genes in the human placenta across gestation. Interestingly, our data reveal that the majority of homeobox gene promoters are hypo-methylated during the first trimester and remain so throughout gestation (Figs 1 to 4 and S1). This indicates that many homeobox gene promoters remain open, allowing the induction of these genes in the placenta at all stages of pregnancy. This may be important, as throughout gestation the placenta must maintain its fundamental roles of active nutrient transport between the maternal and fetal circulations, protecting the fetus from maternal immune rejection, and producing and secreting hormones and growth factors. Homeobox genes have been implicated in regulating these processes. Nevertheless, expression analysis shows that all but two genes have loss of expression over gestation, indicating that expression of homeobox genes peaks in the first trimester (Fig. 6).

We previously showed wide-spread changes in genome-wide promoter methylation in the human placenta over gestation^34^. Overall promoter methylation alone was predictive of gestational age, and several hundred promoters showed significant DNA methylation changes between first and third trimester. A general increase in promoter methylation was observed, suggesting that early gestation is associated with a higher capacity for gene induction. In this study, we sought to investigate the role of DNA methylation in controlling homeobox gene expression in the placenta throughout gestation.

Several homeobox genes became increasingly methylated during gestation, with one or more HM27 probes showing differential methylation. We chose to further explore the role of three genes, DLX5, HOXA10 and TLX1, in placental function, based on significant change in DNA methylation at these genes (Table S1), and an inverse correlation between DNA methylation and level of expression over gestation. The largest DMR was found within the gene body of the homeobox gene *DLX5* (distal-less homeobox 5), with 5 CpG sites within the region showing progressively higher expression in 2^nd^ and 3^rd^ trimesters compared to first trimester villi (Fig. 5). This change in methylation correlated with decreased *DLX5* transcription (Fig. 6A). The specific role of *DLX5* in the human placenta, and the purpose of increased *DLX5* methylation during gestation are not yet known. In a recent study of known and putative imprinted genes in the human placenta, the transcription factor *DLX5* was one of 10 genes that could discern between newborn neurobehavioural profiles deduced from the Network Neurobehavioural Scales^40^. These data suggested that control of *DLX5* expression in the placenta by methylation may be important for neurobehavioural development. Although the relationship between gene body DNA methylation and gene expression control is not always easy to deduce, it is becoming increasingly clear from numerous sources that many gene enhancer elements in the introns of genes can interact with promoters to regulate gene transcription. Based on publically available ENCODE DNAseI tracks, we show that the 1.7 kb region with increased DNA methylation within the DLX5 gene is associated with open chromatin. Therefore, *DLX5* gene body methylation may be located at a regulatory element of the gene, which requires further investigation (Fig. 5).

Another potential role for *DLX5* methylation may be in regulating stem cell function. The murine homologue, *Dlx5*, plays a crucial role in the commitment of bone marrow mesenchymal stem cells (MSCs) into osteoblasts and adipocytes^41^. In the human placenta, we showed that *DLX5* is localised to nuclei of cells within the predicted vascular niche of chorionic mesenchymal stem/stromal cells (CMSC). Furthermore, DLX5 nuclear expression was also detected in cultured first trimester CMSC, and there was a qualitative decrease in DLX5 expression in cultured, term CMSC. Cultured CMSC are capable of differentiating into the mesenchymal lineages including osteoblasts and adipocytes^27^. In a recent study, evidence was provided that *DLX5* is a regulator of human bone marrow MSC osteogenic differentiation and that *DLX5* expression was not associated with a change in DNA methylation level. The authors concluded that the low to partial level of methylation at the CpG island is consistent with the ability of the gene to be induced^42^. Due to the approach used, the authors of that study were only able to interrogate a region of 150 bp, while the distribution of the probes on the HM27 platform allowed us to identify that the DMR is about 1.7 kb in length. At the 5′ end of the CpG Island the methylation level was indeed low and showed only a slight increase in methylation, while at the 3′ end, the methylation change was higher.

Two other genes - *HOXA10* and *TLX1* also showed a significant decrease in RNA expression in second trimester and third trimester placentae compared with first trimester placentae (Fig. 6C), accompanied by higher DNA methylation (Fig. 6D). The concordant change in DNA methylation and gene expression indicated that these genes are regulated during gestation, and may have roles in placental development and function. Next, we determined that within the placental villi, DLX5, HOXA10 and TLX1 protein were localised primarily to the nuclei of syncytiotrophoblast, as well as in stromal cells of both first-trimester and term placentae. Nuclear localisation was consistent with DLX5, HOXA10 and TLX1 acting as nuclear transcription factors (Fig. 7). Furthermore, a decrease in the staining intensity for DLX5, HOXA10 and TLX1 protein was observed using immunohistochemical analysis of term placental tissues compared with first trimester placentae (Fig. 7I). This data, were consistent with the decreased mRNA expression and associated increased DNA methylation across gestation for the candidate genes (Fig. 6C and D). The localisation of these genes to the syncytiotrophoblast later suggests that their decreased expression influences the function of the syncytiotrophoblast, or in the differentiation of trophoblasts.

By using siRNAs specific for *DLX5*, *HOXA10* and *TLX1*, we showed that down-regulation of these genes in primary trophoblasts leads to loss of proliferation, and an increase in mRNA expression of differentiation markers, such as *ERVW-1* (Fig. 8). This suggests that loss of these proteins is required for proper syncytiotrophoblast development during gestation. Interestingly, loss of these genes at the second trimester stage onwards, correlates well with the reported increase in *ERVW-1* expression in the second and third trimester, relative to early pregnancy^43^. In conclusion, our studies demonstrated that the homeobox gene methylation and their transcription are important for normal placental development. Furthermore, it is important to determine whether the increased *DLX5, TLX1* or *HOXA10* methylation and decreased transcription observed during normal placentation are altered in placental pathologies.

## Methods and Materials

### Collection of placental tissues

First trimester placentae (6–12 weeks gestation) were obtained following elective terminations of pregnancy. Term placentae (37–42 weeks gestation) were obtained from uncomplicated pregnancies within 30 min of vaginal or Caesarean delivery. All patients provided written informed consent. Ethical approval was granted by the Committee on Clinical Investigation, Broussais Hospital (Paris, France), North West Local Research Ethical Committee, St. Mary’s Hospital (Manchester, UK), Auckland Medical Aid Centre (AMAC), Auckland (New Zealand) and with the approval from the Northern Health and Disability Ethics Committee (NTX1206067AM05), CYWHS Human Research Ethics Committee (REC2249/2/13) North Adelaide Australia, Monash Health Human Research and Ethics Committee (Clayton, Melbourne) and The Royal Women’s Hospital Human Research and Ethics Committee (Melbourne, Australia) and the study was performed in accordance with the relevant guidelines and regulations. Villous tissue was randomly sampled and snap frozen for RNA and DNA extractions. For immunohistochemical analyses villous tissue was washed in PBS, fixed in neutral buffered formalin (4% (v/v)) for 24 h and embedded in paraffin wax. In total 18 first, 10 second and 14 third trimester villous samples were used for HM27 array analysis. For gene expression and immunohistochemical analysis, 12 first, 4–6 second and 6 third trimester villi preparations were used. For isolation of villous cytotrophoblasts, term placentae were collected from Caesarean delivery of uncomplicated pregnancies (n = 4).

### DNA extraction

DNA was extracted as previously described^34, 44–46^. Tissue samples were incubated at 50 °C overnight with shaking in DNA extraction buffer (100 mM NaCl, 10 mM Tris-HCl (pH 8.0), 25 mM EDTA, 0.5% (w/v) SDS), containing 200 mg/ml proteinase K. DNA was isolated by two rounds of phenol: chloroform extraction, followed by RNAse A treatment. DNA was precipitated in absolute ethanol containing 10% (v/v) sodium acetate (3 M, pH 5.2), and resuspended in 100 ml nuclease-free water (Ambion, Austin, TX, USA). DNA was stored at −20 °C until needed.

### DNA methylation array analysis

We previously performed genome-scale DNA methylation analysis on 18 first (8–12 weeks), 10 second (17–24 weeks) and 14 third trimester placental (34–41 weeks) samples, using the Illumina Infinium Human-Methylation27 BeadChip (HM27)^34^. For this study, data were exported from Gene Expression Omnibus, accession number GSE31781 to analyse the methylation status of homeobox genes. Methylation values for specific homeobox genes were extracted and analysed for significant changes using R (https://cran.r-project.org/) and Excel.

### Gene expression analysis by quantitative real time PCR (RT-PCR)

Relative quantitation of mRNA in placental tissues was performed in an ABI Prism 7500 using Applied Biosystems inventoried assays (Foster City, CA, USA). The assay mix consisted of unlabelled PCR primers and a TaqMan FAM-labelled MGB probes for *DLX5* (Hs01573641_mH); *HOXA10* (Hs00172012_m1); *TLX1* (Hs00271457_m1); *CGB* (Hs01668436_g1); *syncytin-1/ERVW-1* (Hs01926764_u1) and *TP53* (Hs01034249_m1), (Thermo Fisher Scientific, Waltham, MA, USA). Gene expression relative to *18S rRNA* (4319413E, VIC/MGB probe, primer limited, Thermo Fisher Scientific, Waltham, MA, USA) was calculated according to the 2^−ΔΔCT^ method^47^. In total n = 5 first trimester, n = 3 second trimester and n = 2 third trimester samples were profiled for gene expression of a panel of genes. Additionally, DLX5, HOXA10 and TLX1 expression were confirmed in additional n = 12 first, n = 4 second and n = 4 third trimester samples.

### Immunohistochemistry

Sections (5 µm) of wax-embedded placenta were deparaffinised in Histoclear and alcohol and microwaved at full power for 10 min in sodium citrate buffer (0.01 M; containing 0.05% (v/v) Tween 20, pH 6.0) to facilitate antigen unmasking. After cooling for 20 min, sections were washed in distilled water and endogenous peroxide activity was quenched by incubation with 3% (v/v) hydrogen peroxide at room temperature for 10 min. Tissue sections were washed (3 × 5 min) in 0.05 M Tris buffered saline (TBS) and blocked with 5% (w/v) BSA in TBS for 30 min. Primary antibodies, diluted to the required concentration with 5% (w/v) BSA solution [rabbit polyclonal DLX5 EPR4488 antibody 1:500, HOXA10 Ab191470 antibody, 1:750 and TLX1 Ab78498 antibody (Abcam, Cambridge, UK); mouse monoclonal cytokeratin-7 antibody 1:100 (Dako, Glostrup, Denmark) and control rabbit IgG (concentration matched, Sigma-Aldrich, St Louis, MO, USA)] were applied to the tissue sections, which were incubated overnight at 4 °C in a humidity chamber. Slides were washed in TBS (3 × 5 min) and the secondary antibodies, diluted in TBS (biotinylated rabbit anti-goat IgG 1:500; biotinylated goat anti-mouse IgG 1:200, Dako, Glostrup, Denmark), were applied for 30 min at room temperature. Slides were washed again (TBS; 3 × 5 min) and incubated with avidin peroxidase (5 μg/ml; Sigma-Aldrich, St Louis, MO, USA) for 30 min at room temperature. After further TBS washes (3 × 5 min), tissue sections were incubated with 0.05% (w/v) 3,3′-diaminobenzidine (DAB) and 0.015% (v/v) hydrogen peroxide (Sigma-Aldrich, St Louis, MO, USA) and colour development was monitored under a light microscope. Slides were washed in distilled water, counterstained with hematoxylin, rehydrated in alcohol and Histoclear, and mounted in DPX (Sigma-Aldrich, St Louis, MO, USA) for imaging with a Leitz Dialux22 light microscope (Stuggart, Germany). All tissue sections were incubated with DAB for the same length of time so that comparisons could be made between individual samples, and all slides were stained in a single run to eliminate inter-experimental variations in staining intensity. Exposure times and background colour were matched at image capture. The intensity of staining was calculated using the AxioVision software (Carl Zeiss, Oberkochen, Germany) from five fields of view of each placental section and the results are expressed as the mean pixel density (in arbitrary units).

### Villous cytotrophoblast isolation, culture and siRNA treatment

Villous cytotrophoblasts (VCTs) were prepared from placentae obtained from uncomplicated term pregnancies as described previously^31^. Briefly, VCTs were enriched by differential sequential trypsin digestion and the use of a Percoll gradient (20–60%). VCTs were plated at a density of 1.0 × 10^4^ cells/cm^2^ in 6-well tissue culture plates using DMEM supplemented with 10% fetal bovine serum (FBS), 2 mM glutamine, 100 µg/mL streptomycin, 100 IU/mL penicillin. The purity of VCT cultures was characterised by positive staining for CK7 (95% positive cells) and by microscopic observation of cell aggregates and syncytiotrophoblast formation at 48 to 72 hours. VCT were seeded at a density of 0.5 × 10^5^ cells/well in 24-well plates and transfected with Thermo Fisher Scientific (Waltham, MA, USA) DLX5 siRNA (siDLX5) or HOXA10 siRNA (siHOXA10) or TLX1 siRNA (siTLX1), using the HiPerFect transfection reagent (Qiagen, Hilden, Germany). The optimum siRNA: HiPerFect ratio and incubation time for the culture was determined to be 1:6 and 72 h, respectively (data not shown). Negative control siRNA (siCONT) used in these experiments consisted of an enzyme-generated pool of oligonucleotides (15–19 base pairs) that showed no DNA sequence similarity to any known human gene (AllStars Neg. siRNA AF 488, Qiagen, Hilden, Germany)^31^. At the end of the incubation, the cells were collected for analysis of gene expression for *DLX5, HOXA10, TLX1*, and trophoblast differentiation markers *CGB*, *ERVW-1* and apoptosis marker, *TP53*.

### Cell proliferation assay

VCTs were grown in 96-well plate (2 × 10^4^ cells/well, Corning) for 24 h. Following siDLX5/siHOXA10/siTLX1 or siCONT treatment of trophoblasts, the proliferation potential of cells was determined using the CellTiter 96® AQueous One Solution Cell Proliferation assay (Promega Corporation, Madison, WI, USA) and the absorbance at 490 nm was recorded using an ELISA plate reader (SpectraMax i3).

### Statistical analyses

A p-value of <0.05 was considered statistically significant. The LIMMA package was used for HM27 DNA methylation analysis, and probes were considered significant based on an adjusted p-value (Benjamini correction).

## Electronic supplementary material

Supplementary figures and legends

## References

[1] Morrish DW, Dakour J, Li H (2001). Life and death in the placenta: new peptides and genes regulating human syncytiotrophoblast and extravillous cytotrophoblast lineage formation and renewal. Curr Protein Pept Sci.

[2] Knofler M (2005). Trophoblast signalling: knowns and unknowns–a workshop report. Placenta.

[3] Knofler M, Vasicek R, Schreiber M (2001). Key regulatory transcription factors involved in placental trophoblast development–a review. Placenta.

[4] Knofler M, Pollheimer J (2013). Human placental trophoblast invasion and differentiation: a particular focus on Wnt signaling. Front Genet.

[5] Cross JC (2000). Genetic insights into trophoblast differentiation and placental morphogenesis. Semin Cell Dev Biol.

[6] Latos PA, Hemberger M (2014). Review: the transcriptional and signalling networks of mouse trophoblast stem cells. Placenta.

[7] Boncinelli E (1999). Homeobox genes in development. Adv Neurol.

[8] Gehring WJ (1985). Homeotic genes, the homeobox, and the spatial organization of the embryo. Harvey Lect.

[9] Nunes FD, de Almeida FC, Tucci R, de Sousa SC (2003). Homeobox genes: a molecular link between development and cancer. Pesqui Odontol Bras.

[10] Boncinelli E (1989). Organization of human class I homeobox genes. Genome.

[11] Gehring WJ, Hiromi Y (1986). Homeotic genes and the homeobox. Annu Rev Genet.

[12] Akam M (1998). Hox genes: from master genes to micromanagers. Curr Biol.

[13] Lohmann I, McGinnis W (2002). Hox Genes: it’s all a matter of context. Curr Biol.

[14] Mann RS (1997). Why are Hox genes clustered?. BioEssays.

[15] Acampora D, Pannese M, D’Esposito M, Simeone A, Boncinelli E (1987). Human homoeobox-containing genes in development. Hum Reprod.

[16] Oudejans CB, Pannese M, Simeone A, Meijer CJ, Boncinelli E (1990). The three most downstream genes of the Hox-3 cluster are expressed in human extraembryonic tissues including trophoblast of androgenetic origin. Development.

[17] Chui A (2010). Homeobox gene distal-less 3 is expressed in proliferating and differentiating cells of the human placenta. Placenta.

[18] Chui A (2012). Homeobox gene Distal-less 3 is a regulator of villous cytotrophoblast differentiation and its expression is increased in human idiopathic foetal growth restriction. J Mol Med (Berl).

[19] Grati FR (2004). Biparental expression of ESX1L gene in placentas from normal and intrauterine growth-restricted pregnancies. Eur J Hum Genet.

[20] Murthi P (2006). Homeobox gene HLX1 expression is decreased in idiopathic human fetal growth restriction. Am J Pathol.

[21] Murthi P (2006). Homeobox gene ESX1L expression is decreased in human pre-term idiopathic fetal growth restriction. Mol Hum Reprod.

[22] Murthi P (2006). Homeobox gene DLX4 expression is increased in idiopathic human fetal growth restriction. Mol Hum Reprod.

[23] Murthi P (2007). Homeobox genes are differentially expressed in macrovascular human umbilical vein endothelial cells and microvascular placental endothelial cells. Placenta.

[24] Quinn LM, Johnson BV, Nicholl J, Sutherland GR, Kalionis B (1997). Isolation and identification of homeobox genes from the human placenta including a novel member of the Distal-less family, DLX4. Gene.

[25] Rajaraman G, Murthi P, Quinn L, Brennecke SP, Kalionis B (2008). Homeodomain protein HLX is expressed primarily in cytotrophoblast cell types in the early pregnancy human placenta. Reprod Fertil Dev.

[26] Sun YY (2011). Regulation of epithelial-mesenchymal transition by homeobox gene DLX4 in JEG-3 trophoblast cells: a role in preeclampsia. Reprod Sci.

[27] Liu H (2014). A novel combination of homeobox genes is expressed in mesenchymal chorionic stem/stromal cells in first trimester and term pregnancies. Reprod Sci.

[28] Rajaraman G, Murthi P, Brennecke SP, Kalionis B (2010). Homeobox gene HLX is a regulator of HGF/c-met-mediated migration of human trophoblast-derived cell lines. Biol Reprod.

[29] Rajaraman G, Murthi P, Pathirage N, Brennecke SP, Kalionis B (2010). Downstream targets of homeobox gene HLX show altered expression in human idiopathic fetal growth restriction. Am J Pathol.

[30] Sun Y, Lu X, Yin L, Zhao F, Feng Y (2006). Inhibition of DLX4 promotes apoptosis in choriocarcinoma cell lines. Placenta.

[31] Chui A (2013). Downstream targets of the homeobox gene DLX3 are differentially expressed in the placentae of pregnancies affected by human idiopathic fetal growth restriction. Mol Cell Endocrinol.

[32] Chui A (2011). Homeobox gene Distal-less 3 (DLX3) is a regulator of villous cytotrophoblast differentiation. Placenta.

[33] Murthi P, Abumaree M, Kalionis B (2014). Analysis of homeobox gene action may reveal novel angiogenic pathways in normal placental vasculature and in clinical pregnancy disorders associated with abnormal placental angiogenesis. Front Pharmacol.

[34] Novakovic B (2011). Evidence for widespread changes in promoter methylation profile in human placenta in response to increasing gestational age and environmental/stochastic factors. BMC Genomics.

[35] Januar V, Desoye G, Novakovic B, Cvitic S, Saffery R (2015). Epigenetic regulation of human placental function and pregnancy outcome: considerations for causal inference. Am J Obstet Gynecol.

[36] Tsumagari K (2013). DNA methylation and differentiation: HOX genes in muscle cells. Epigenetics Chromatin.

[37] Avraham A (2010). A similar cell-specific pattern of HOXA methylation in normal and in cancer tissues. Epigenetics.

[38] Illingworth R (2008). A novel CpG island set identifies tissue-specific methylation at developmental gene loci. PLoS Biol.

[39] Blair JD, Langlois S, McFadden DE, Robinson WP (2014). Overlapping DNA methylation profile between placentas with trisomy 16 and early-onset preeclampsia. Placenta.

[40] Green BB (2015). Expression of imprinted genes in placenta is associated with infant neurobehavioral development. Epigenetics.

[41] Baek K, Baek JH (2013). The transcription factors myeloid elf-1-like factor (MEF) and distal-less homeobox 5 (Dlx5) inversely regulate the differentiation of osteoblasts and adipocytes in bone marrow. Adipocyte.

[42] Farshdousti Hagh M (2015). Different Methylation Patterns of RUNX2, OSX, DLX5 and BSP in Osteoblastic Differentiation of Mesenchymal Stem Cells. Cell J.

[43] Chen CP (2006). Altered placental syncytin and its receptor ASCT2 expression in placental development and pre-eclampsia. BJOG.

[44] Novakovic B (2008). Specific tumour-associated methylation in normal human term placenta and first-trimester cytotrophoblasts. Mol Hum Reprod.

[45] Novakovic B (2009). Placenta-specific methylation of the vitamin D 24-hydroxylase gene: implications for feedback autoregulation of active vitamin D levels at the fetomaternal interface. J Biol Chem.

[46] Ollikainen M (2010). DNA methylation analysis of multiple tissues from newborn twins reveals both genetic and intrauterine components to variation in the human neonatal epigenome. Hum Mol Genet.

[47] Livak KJ, Schmittgen TD (2001). Analysis of relative gene expression data using real-time quantitative PCR and the 2(-Delta Delta C(T)) Method. Methods.

