# Supplementary material for: Increased methylation and decreased expression of homeobox genes TLX1, HOXA10 and DLX5 in human placenta are associated with trophoblast differentiation
Source: Sci Rep. 2017 Jul 3;7:4523. doi: 10.1038/s41598-017-04776-5 (PMC5495813; doi:10.1038/s41598-017-04776-5)
Supplement: Supplementary file 1 — Supplementary figures and legends [file 41598_2017_4776_MOESM1_ESM.pdf]

Increased methylation and decreased expression of homeobox genes *TLX1, HOXA10* and *DLX5* in human placenta are associated with trophoblast differentiation.

Boris Novakovic[a], Thierry Fournier[b,c,d], Lynda K Harris[e], Joanna James[f], Claire T Roberts[g], Hannah EJ Yong[h], Bill Kalionis[h], Danièle Evain-Brion[b,c,d], Peter R Ebeling[i], Euan M Wallace[j,k], Richard Saffery[a,l], Padma Murthi[h,i,j]*

[a]Cancer and Disease Epigenetics, Murdoch Childrens Research Institute, Royal Children's Hospital, Victoria, Australia. [b]INSERM, UMR-S1139, Faculté des Sciences Pharmaceutiques et Biologiques, Paris, France. [c]Université Paris Descartes, Paris, France. [d]Fondation PremUp, Paris, France. [e]Manchester Pharmacy School, The University of Manchester, Manchester, UK. [f]Department of Obstetrics and Gynaecology, University of Auckland, New Zealand. [g]Robinson Research Institute, and Adelaide Medical School, University of Adelaide, South Australia. [h]Department of Maternal-Fetal Medicine Pregnancy Research Centre, The Royal Women's Hospital, and Department of Obstetrics and Gynaecology, University of Melbourne, Victoria, Australia. [i]Department of Medicine, School of Clinical Sciences, Monash University, Victoria, Australia. [j]The Ritchie Centre, Hudson Institute of Medical Research, Victoria, Australia. [k]Department of Obstetrics and Gyaecology, Monash University, Victoria, Australia. [l]Department of Paediatrics, University of Melbourne, Victoria, Australia

* Corresponding author

Dr Padma Murthi: Email: padma.murthi@monash.edu

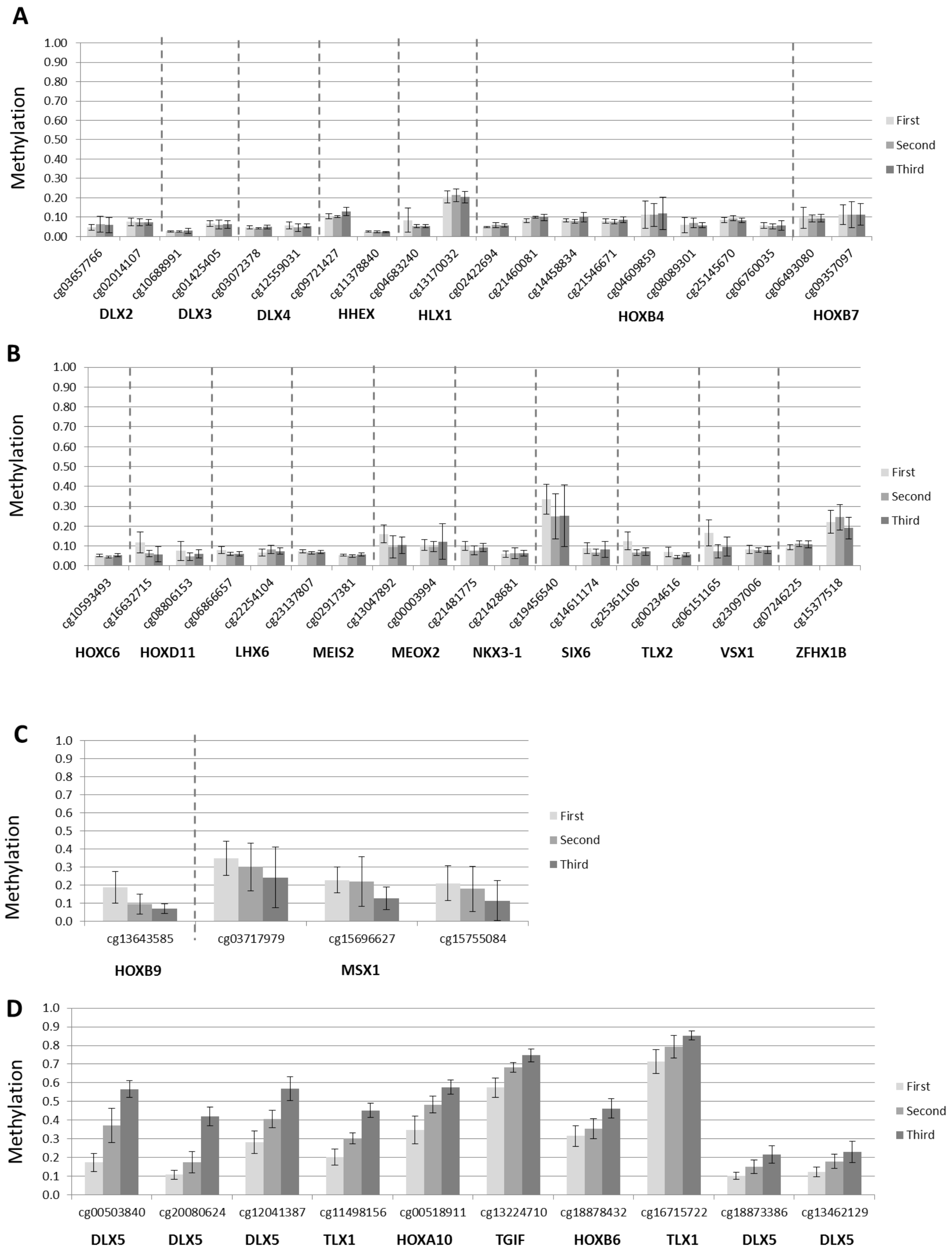

Figure S1 Homeobox genes showing stable methylation over gestation or dynamic methylation over gestation. (A) Most HM27K array probes that were assigned to homeobox genes showed

consistent DNA methylation over gestation. For the majority of sites, DNA methylation level was low, as expected for promoter regions. DNA methylation is shown for each site per homeobox gene, with first trimester shown in light grey, second in dark grey, and third trimester in black. (B-C) DNA methylation levels over gestation are shown for the nine genes that showed dynamic changes. Panel B shows genes with decreased methylation over gestation while panel C shows genes with increased methylation over gestation. *DLX5* had 5 CpG sites that showed increase in DNA methylation over gestation. DNA methylation is shown for each site per homeobox gene, with first trimester shown in light grey, second in dark grey, and third trimester in black.

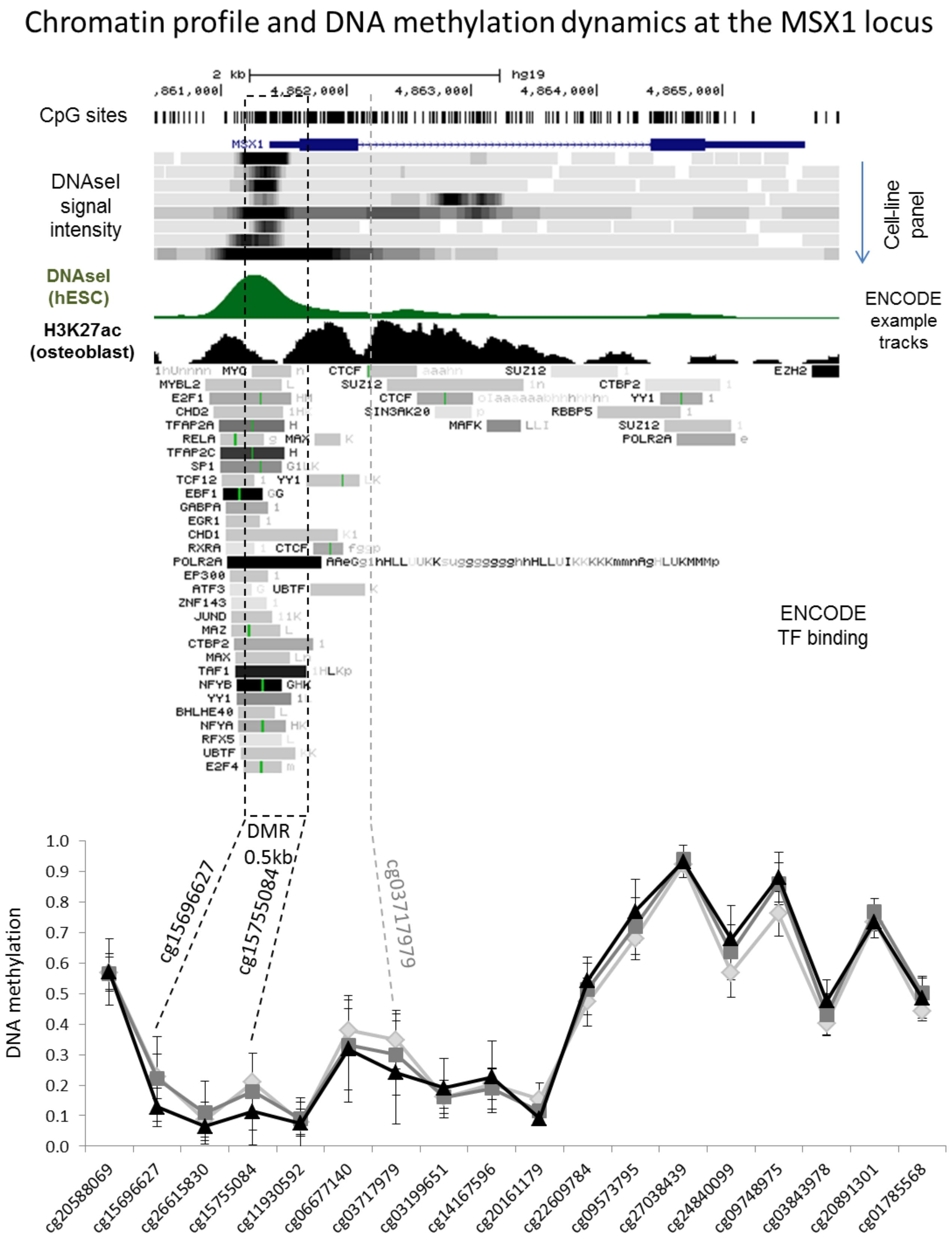

Figure S2 ***MSX1*** **promoter region differential methylation.** *MSX1* has 3 HM27 probes with dynamic DNA methylation over gestation. By plotting DNA methylation in first, second and third

trimester for each site at this gene, it is clear that DNA methylation change is occurring in the promoter region of the gene. ENCODE DNAseI track shows that the DMR is associated with open chromatin in a subset of human tissues, indicating that change in DNA methylation may result in change of accessibility.

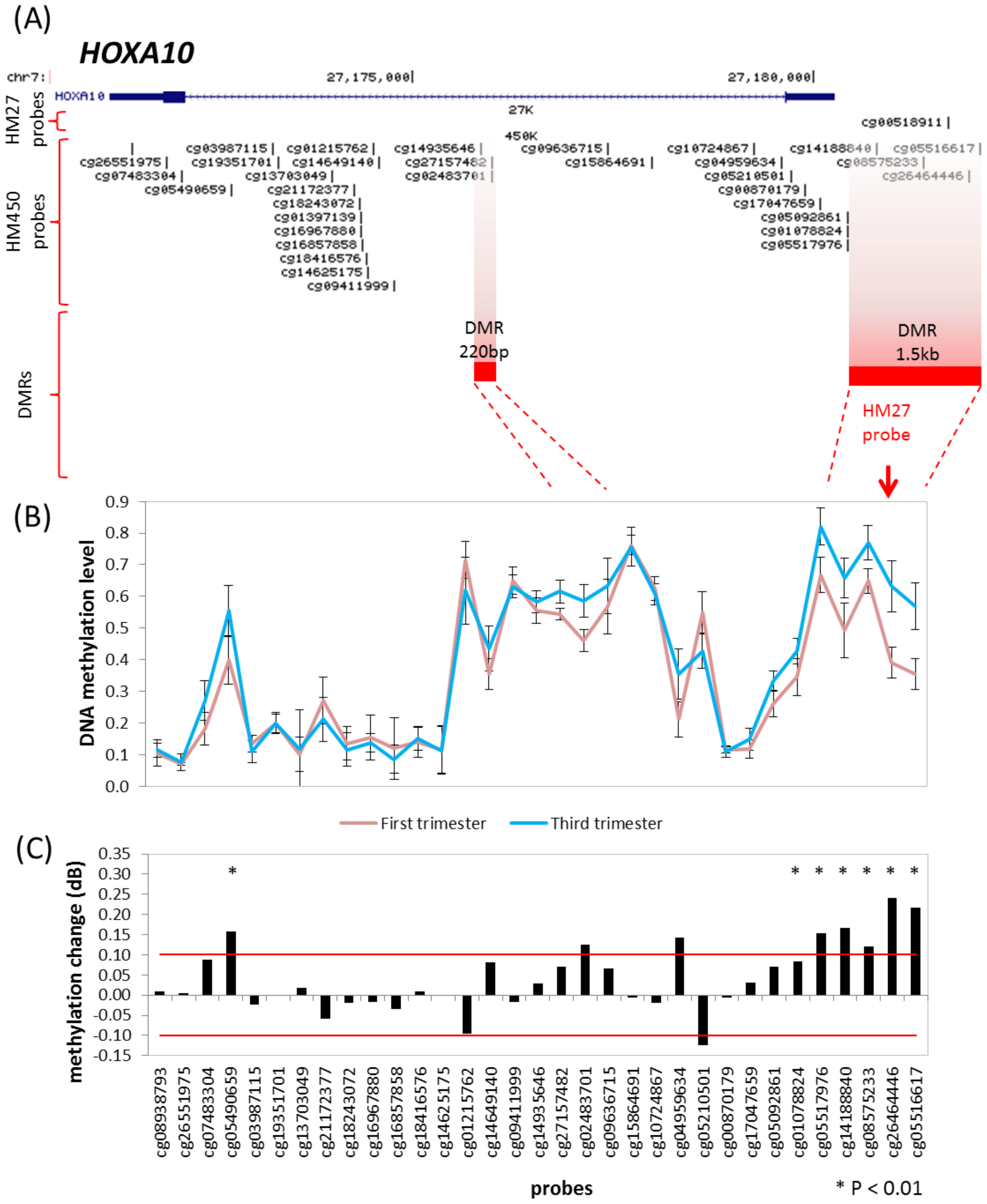

Figure S3 DNA methylation around the HOXA10 gene. Infinium HM450 array data was used from Blair et al. 2014. (A) location of HOXA10 gene and HM27 and HM450 probe locations. In total, 33 CpG probes cover the HOXA10 locus, which is an increase from 1 CpG probe from the HM27 array (location of which is indicated with red arrow). (B) Average methylation level for all

HM450 probes in first and third trimester placental villi. Data is shown for of n=5 first trimester and n=10 third trimester placental villi, and error bars are standard deviation. (C) Change in methylation between first and third trimester (positive value = increase over time, negative value = decrease over time). The original differential HM27 probe is surrounded by five HM450 probes that also increase in methylation over gestation, indicating that this region is a differentially methylated region (DMR).

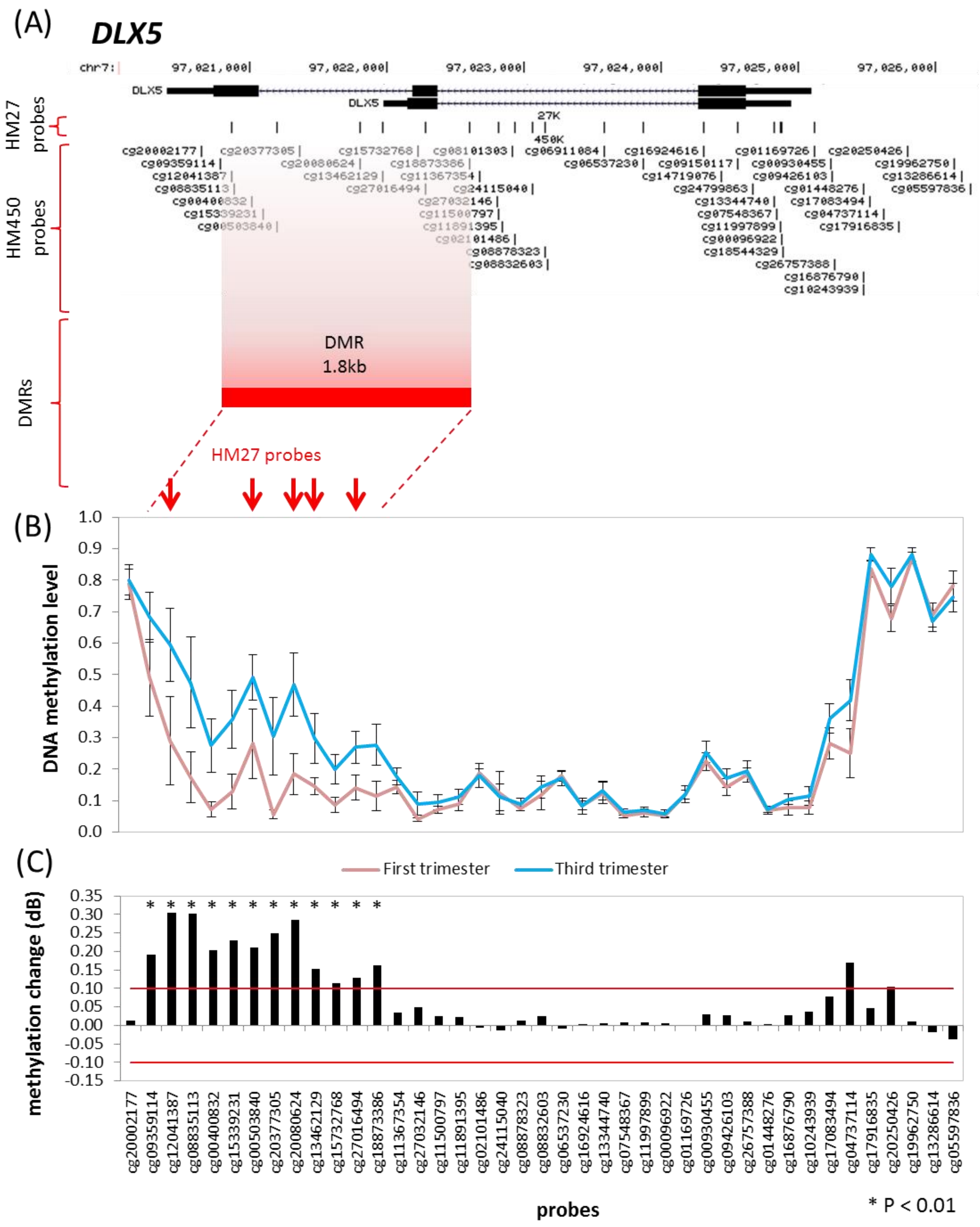

Figure S4 DNA methylation around the DLX5 gene. Infinium HM450 array data was used from Blair et al. 2014. (A) location of DLX5 gene and HM27 and HM450 probe locations. In total, 41 CpG probes cover the DLX5 locus, which is an increase from 18 CpG probes from the HM27 array (location of which is indicated with red arrow). (B) Average methylation level for all HM450 probes

in first and third trimester placental villi. Data is shown for of n=5 first trimester and n=10 third trimester placental villi, and error bars are standard deviation. (C) Change in methylation between first and third trimester (positive value = increase over time, negative value = decrease over time). The original five differential HM27 probes are in a region with twelve HM450 probes that also increase in methylation over gestation, indicating that this region is a differentially methylated region (DMR).

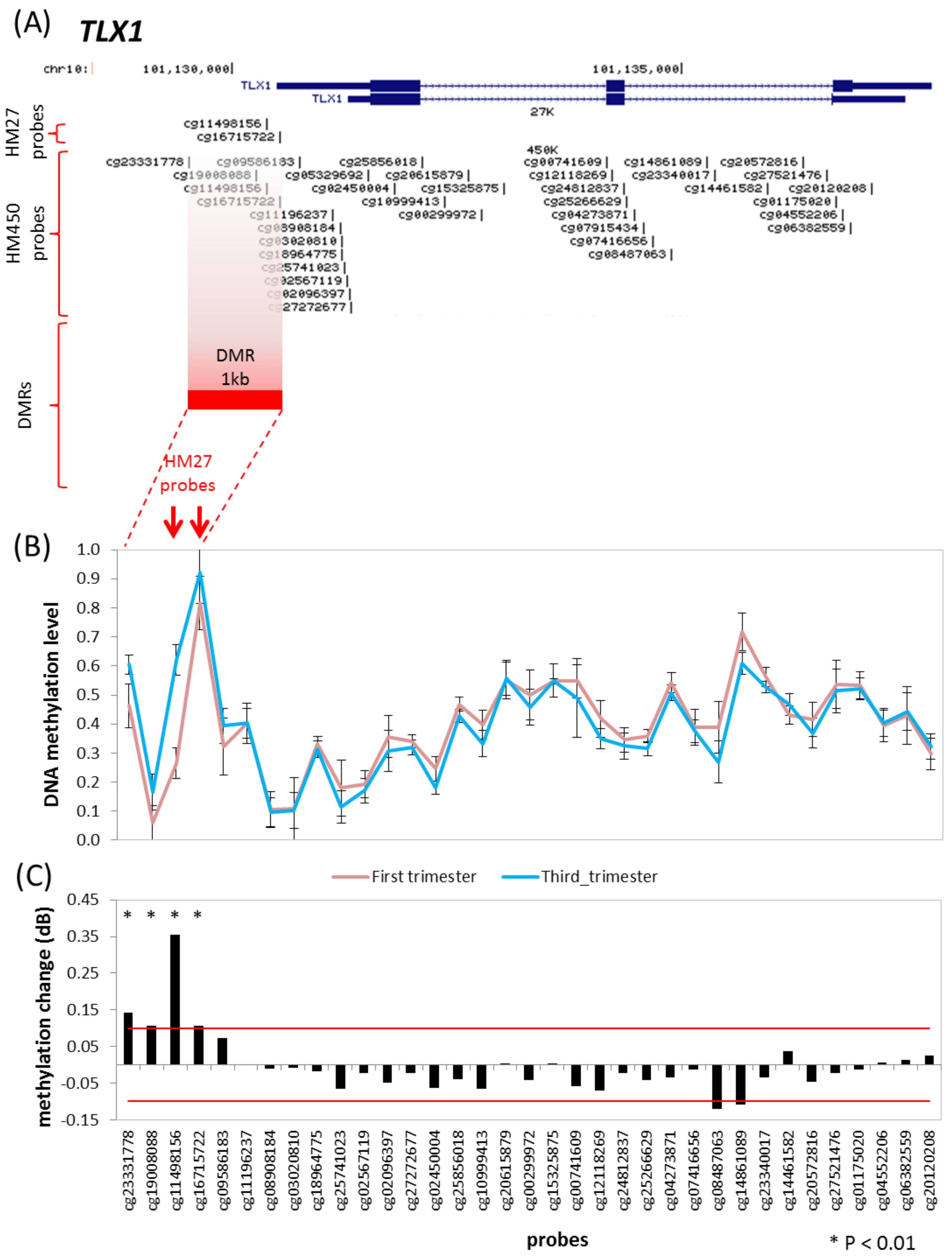

Figure S5 DNA methylation around the TLX1 gene. Infinium HM450 array data was used from Blair et al. 2014. (A) location of TLX1 gene and HM27 and HM450 probe locations. In total, 35

CpG probes cover the TLX1 locus, which is an increase from 2 CpG probes from the HM27 array (location of which is indicated with red arrow). (B) Average methylation level for all HM450 probes in first and third trimester placental villi. Data is shown for of n=5 first trimester and n=10 third trimester placental villi, and error bars are standard deviation. (C) Change in methylation between first and third trimester (positive value = increase over time, negative value = decrease over time). The original two differential HM27 probes are in a region with four HM450 probes that also increase in methylation over gestation, indicating that this region is a differentially methylated region (DMR).

Table S1. List of HOX-associated probes, ranked by p-value based on first trimester (n=18) and third trimester (n=14) methylation.

Symbol	ID	1st trim	1st sd	2nd trim	2nd sd	3rd trim	Term sd	- logPvalue	Functional analysis?
DLX5	cg00503840	0.17	0.05	0.37	0.09	0.57	0.05	20.220	Yes
DLX5	cg20080624	0.11	0.02	0.17	0.06	0.42	0.05	20.053	Yes
TLX1	cg11498156	0.20	0.04	0.30	0.03	0.45	0.04	16.324	yes
DLX5	cg12041387	0.28	0.06	0.41	0.05	0.57	0.06	13.187	Yes
HOXB3	cg12910797	0.41	0.08	0.55	0.07	0.69	0.05	11.510	
TGIF	cg13224710	0.57	0.05	0.68	0.02	0.75	0.03	11.096	
HOXA10	cg00518911	0.35	0.07	0.48	0.04	0.58	0.04	10.793	Yes
DLX5	cg18873386	0.10	0.02	0.15	0.04	0.22	0.05	9.934	
ALX4	cg14144305	0.34	0.05	0.25	0.08	0.15	0.08	8.505	
TLX1	cg16715722	0.71	0.06	0.79	0.06	0.85	0.02	8.085	
HOXB6	cg18878432	0.32	0.06	0.35	0.05	0.46	0.05	7.960	
HOXB1	cg07823492	0.57	0.10	0.73	0.07	0.80	0.06	7.503	
DLX5	cg27016494	0.10	0.02	0.16	0.06	0.19	0.05	7.268	
DLX5	cg13462129	0.12	0.03	0.18	0.04	0.23	0.06	7.182	
ALX4	cg04970352	0.48	0.06	0.38	0.11	0.26	0.12	6.983	
ALX4	cg00418150	0.60	0.04	0.55	0.05	0.70	0.04	6.796	
HOXA11	cg17950095	0.35	0.06	0.26	0.09	0.15	0.13	5.950	
HOXA9	cg01354473	0.36	0.08	0.47	0.07	0.53	0.08	5.746	
ALX4	cg00415164	0.15	0.04	0.12	0.08	0.08	0.01	5.425	
HOXA9	cg01381846	0.38	0.09	0.47	0.11	0.52	0.07	4.937	
ALX4	cg04549333	0.25	0.07	0.19	0.07	0.12	0.06	4.884	
HOXB2	cg25882366	0.34	0.09	0.36	0.09	0.49	0.08	4.879	
HOXB9	cg13643585	0.19	0.09	0.09	0.05	0.07	0.03	4.568	
HOXC4	cg21487207	0.45	0.06	0.30	0.05	0.29	0.13	4.360	
ALX4	cg13807496	0.14	0.03	0.08	0.03	0.09	0.03	4.244	
ALX4	cg15522957	0.49	0.07	0.52	0.05	0.63	0.09	4.244	
HOXA9	cg27009703	0.50	0.07	0.58	0.03	0.61	0.07	4.089	
ALX4	cg19358442	0.29	0.08	0.27	0.08	0.17	0.08	3.879	
HOXB4	cg02422694	0.05	0.00	0.06	0.01	0.06	0.01	3.870	
MSX1	cg09748975	0.76	0.07	0.86	0.07	0.88	0.08	3.814	
MSX1	cg20161179	0.15	0.05	0.12	0.03	0.09	0.02	3.786	
TLX2	cg25361106	0.13	0.04	0.07	0.01	0.07	0.02	3.721	
MSX1	cg15696627	0.23	0.07	0.22	0.14	0.13	0.06	3.584	
ALX4	cg08914623	0.13	0.07	0.07	0.03	0.04	0.03	3.461	
ALX4	cg08993172	0.16	0.04	0.13	0.02	0.11	0.04	3.302	
HHEX	cg09721427	0.11	0.01	0.10	0.01	0.13	0.02	3.236	
MSX1	cg03843978	0.40	0.04	0.43	0.07	0.48	0.07	3.222	
TGIF	cg20322862	0.17	0.03	0.13	0.03	0.13	0.03	3.045	
HOXB4	cg21460081	0.08	0.01	0.10	0.01	0.10	0.02	3.006	
MEOX2	cg13047892	0.16	0.04	0.10	0.06	0.10	0.04	2.962	
HOXD11	cg16632715	0.12	0.05	0.06	0.02	0.06	0.04	2.910	
LHX6	cg06866657	0.08	0.02	0.06	0.01	0.06	0.01	2.749	
DLX5	cg09150117	0.08	0.02	0.11	0.04	0.10	0.02	2.617	
MSX1	cg24840099	0.57	0.08	0.64	0.09	0.68	0.11	2.609	
VSX1	cg06151165	0.16	0.07	0.07	0.03	0.10	0.05	2.563	
HOXA13	cg10883303	0.07	0.04	0.05	0.03	0.04	0.01	2.276	
HOXC12	cg05589246	0.14	0.05	0.11	0.02	0.10	0.03	2.256	
HOXB5	cg01405107	0.10	0.01	0.09	0.01	0.11	0.02	2.250	
ZFHX1B	cg07246225	0.09	0.01	0.11	0.01	0.11	0.02	2.201	
MSX1	cg09573795	0.68	0.07	0.72	0.09	0.77	0.10	2.138	
HOXB8	cg25928579	0.16	0.03	0.16	0.04	0.13	0.04	2.128	
HOXC11	cg07123069	0.22	0.06	0.12	0.07	0.15	0.09	2.114	
DLX5	cg08878323	0.10	0.02	0.11	0.03	0.12	0.02	1.965	
MSX1	cg15755084	0.21	0.10	0.18	0.13	0.11	0.11	1.885	
HOXB6	cg16848873	0.16	0.03	0.15	0.03	0.13	0.03	1.879	
MSX1	cg22609784	0.47	0.08	0.51	0.08	0.54	0.08	1.835	
HOXD3	cg18702197	0.22	0.05	0.16	0.09	0.16	0.07	1.795	
ALX4	cg26365854	0.07	0.03	0.05	0.01	0.05	0.01	1.779	
HOXA7	cg26511321	0.71	0.05	0.64	0.06	0.75	0.04	1.778	
ALX4	cg11260848	0.35	0.06	0.30	0.06	0.27	0.13	1.734	
HOXA1	cg12686016	0.14	0.07	0.10	0.02	0.09	0.02	1.702	
HOXC11	cg22709192	0.32	0.07	0.23	0.07	0.25	0.09	1.677	
HOXB2	cg09313705	0.74	0.04	0.70	0.05	0.78	0.06	1.674	
MSX1	cg01785568	0.44	0.03	0.50	0.05	0.48	0.07	1.664	
HOXD9	cg10957151	0.22	0.10	0.15	0.05	0.15	0.06	1.607	
HOXD12	cg03874199	0.34	0.07	0.27	0.09	0.27	0.12	1.594	
HOXC12	cg19928450	0.17	0.04	0.14	0.04	0.13	0.05	1.589	
HOXA11	cg15760840	0.16	0.04	0.10	0.04	0.12	0.05	1.572	
HOXB4	cg14458834	0.08	0.01	0.08	0.01	0.10	0.03	1.523	
HOXC5	cg15700739	0.23	0.02	0.19	0.03	0.25	0.05	1.521	
DLX5	cg11500797	0.08	0.02	0.09	0.01	0.10	0.02	1.520	
MSX1	cg03717979	0.35	0.10	0.30	0.13	0.24	0.17	1.505	
HOXD10	cg03918304	0.30	0.04	0.39	0.07	0.38	0.15	1.494	
TLX2	cg00234616	0.07	0.02	0.04	0.01	0.06	0.01	1.386	
HOXC8	cg25917510	0.05	0.01	0.03	0.00	0.04	0.00	1.343	
ALX4	cg15817236	0.10	0.03	0.07	0.02	0.07	0.03	1.336	
SIX6	cg19456540	0.34	0.08	0.25	0.11	0.25	0.16	1.282	
DLX5	cg16924616	0.05	0.01	0.06	0.01	0.05	0.01	1.271	
HHEX	cg11378840	0.03	0.00	0.02	0.00	0.02	0.00	1.250	

Symbol	ID	1st trim	1st sd	2nd trim	2nd sd	3rd trim	Term sd	- logPvalue	Functional analysis?
HOXA2	cg09871315	0.32	0.07	0.22	0.07	0.26	0.10	**1.247**	
HOXC5	cg16983211	0.12	0.01	0.13	0.01	0.13	0.01	**1.236**	
DLX5	cg00096922	0.06	0.01	0.06	0.01	0.06	0.01	**1.223**	
HOXC10	cg26153631	0.20	0.04	0.18	0.03	0.23	0.05	**1.210**	
HOXA9	cg26521404	0.54	0.10	0.59	0.06	0.60	0.09	**1.198**	
HOXA5	cg12128839	0.05	0.01	0.06	0.01	0.06	0.01	**1.154**	
ALX4	cg14753432	0.48	0.03	0.48	0.05	0.50	0.04	**1.143**	
ALX4	cg27318318	0.09	0.02	0.06	0.01	0.08	0.02	**1.115**	
ALX4	cg00061629	0.35	0.08	0.49	0.09	0.42	0.13	**1.071**	
HLX1	cg04683240	0.08	0.06	0.05	0.01	0.05	0.01	**1.039**	
HOXB4	cg21546671	0.08	0.01	0.08	0.01	0.09	0.02	**0.986**	
HOXB5	cg16495265	0.23	0.06	0.18	0.06	0.18	0.11	**0.946**	
HOXC4	cg10918927	0.04	0.01	0.03	0.00	0.03	0.01	**0.940**	
ALX4	cg23058901	0.47	0.04	0.47	0.04	0.51	0.08	**0.929**	
HOXC9	cg21272774	0.08	0.03	0.10	0.02	0.10	0.02	**0.927**	
ZFHX1B	cg15377518	0.22	0.06	0.25	0.06	0.19	0.05	**0.888**	
HOXA4	cg24169822	0.56	0.06	0.54	0.10	0.60	0.09	**0.874**	
HOXB8	cg15539420	0.33	0.08	0.30	0.11	0.26	0.17	**0.867**	
HOXD10	cg21591742	0.26	0.06	0.20	0.08	0.22	0.09	**0.847**	
DLX5	cg13344740	0.18	0.01	0.15	0.02	0.19	0.02	**0.833**	
ALX4	cg18546622	0.27	0.07	0.31	0.06	0.31	0.10	**0.833**	
HOXA9	cg25047280	0.33	0.07	0.32	0.10	0.40	0.17	**0.828**	
MEIS2	cg23137807	0.07	0.01	0.07	0.01	0.07	0.01	**0.827**	
HOXA5	cg02248486	0.10	0.03	0.11	0.03	0.12	0.05	**0.803**	
HOXD9	cg14991487	0.10	0.07	0.06	0.03	0.06	0.03	**0.791**	
MSX1	cg06677140	0.38	0.07	0.33	0.15	0.32	0.17	**0.739**	
HOXD4	cg00767581	0.33	0.07	0.34	0.08	0.29	0.08	**0.729**	
HOXC13	cg20587394	0.04	0.01	0.04	0.01	0.04	0.01	**0.708**	
HOXD4	cg12127282	0.41	0.07	0.45	0.11	0.45	0.13	**0.664**	
DLX2	cg03657766	0.05	0.02	0.06	0.04	0.06	0.04	**0.642**	
ALX4	cg04988423	0.04	0.01	0.03	0.01	0.03	0.01	**0.637**	
MSX1	cg26615830	0.09	0.06	0.11	0.10	0.06	0.05	**0.629**	
HOXD11	cg08806153	0.08	0.05	0.05	0.02	0.06	0.02	**0.617**	
LHX6	cg22254104	0.07	0.02	0.08	0.02	0.07	0.02	**0.616**	
MSX1	cg03199651	0.16	0.04	0.16	0.05	0.19	0.10	**0.607**	
DLX5	cg02101486	0.15	0.03	0.18	0.03	0.17	0.04	**0.602**	
MEIS2	cg02917381	0.05	0.00	0.05	0.01	0.06	0.01	**0.573**	
HOXB13	cg15786837	0.13	0.04	0.11	0.04	0.11	0.06	**0.549**	
NKX3-1	cg21481775	0.10	0.02	0.08	0.02	0.09	0.02	**0.521**	
HOXC10	cg08900043	0.03	0.01	0.03	0.01	0.03	0.01	**0.476**	
HOXB9	cg12370791	0.03	0.01	0.03	0.00	0.03	0.01	**0.476**	
ALX4	cg16977035	0.45	0.06	0.43	0.07	0.47	0.06	**0.448**	
HOXD4	cg14399060	0.12	0.04	0.11	0.02	0.11	0.04	**0.445**	
DLX3	cg10688991	0.03	0.00	0.03	0.00	0.03	0.01	**0.437**	
DLX3	cg01425405	0.07	0.01	0.06	0.02	0.06	0.02	**0.436**	
ALX4	cg25363445	0.27	0.05	0.25	0.04	0.25	0.06	**0.433**	
HOXA1	cg03700462	0.05	0.01	0.05	0.01	0.06	0.04	**0.430**	
HOXD4	cg01152019	0.12	0.04	0.11	0.04	0.11	0.02	**0.381**	
MSX1	cg14167596	0.20	0.05	0.19	0.07	0.23	0.12	**0.356**	
DLX5	cg01169726	0.08	0.02	0.05	0.01	0.07	0.02	**0.354**	
MEOX2	cg00003994	0.11	0.03	0.10	0.03	0.12	0.09	**0.352**	
NKX3-1	cg21428681	0.06	0.02	0.06	0.03	0.06	0.01	**0.324**	
HOXA6	cg04265576	0.33	0.07	0.26	0.10	0.37	0.18	**0.323**	
ALX4	cg11797994	0.34	0.03	0.31	0.02	0.33	0.03	**0.315**	
MSX1	cg27038439	0.92	0.02	0.94	0.01	0.93	0.05	**0.291**	
DLX5	cg24115040	0.08	0.02	0.09	0.01	0.09	0.01	**0.273**	
DLX5	cg06537230	0.11	0.03	0.12	0.06	0.12	0.03	**0.262**	
HOXC9	cg26829131	0.06	0.02	0.06	0.02	0.06	0.02	**0.257**	
DLX5	cg06911084	0.09	0.05	0.11	0.05	0.11	0.07	**0.256**	
HOXB13	cg21842478	0.05	0.01	0.05	0.00	0.05	0.01	**0.252**	
HOXA7	cg23432345	0.08	0.03	0.07	0.02	0.09	0.06	**0.233**	
DLX4	cg03072378	0.05	0.01	0.04	0.00	0.05	0.01	**0.232**	
HOXB1	cg17233506	0.60	0.05	0.59	0.04	0.61	0.05	**0.228**	
SIX6	cg14611174	0.09	0.03	0.07	0.01	0.08	0.04	**0.226**	
DLX4	cg12559031	0.06	0.02	0.05	0.02	0.06	0.01	**0.219**	
DLX5	cg11997899	0.22	0.07	0.27	0.06	0.23	0.03	**0.194**	
VSX1	cg23097006	0.08	0.02	0.08	0.01	0.08	0.02	**0.165**	
DLX2	cg02014107	0.08	0.02	0.07	0.02	0.07	0.01	**0.162**	
HOXA9	cg07778029	0.20	0.09	0.18	0.07	0.21	0.11	**0.161**	
ALX4	cg07696033	0.09	0.03	0.09	0.02	0.10	0.06	**0.142**	
HOXD8	cg15520279	0.03	0.00	0.02	0.00	0.03	0.01	**0.138**	
HOXD13	cg07175883	0.03	0.00	0.03	0.00	0.03	0.01	**0.128**	
HOXA4	cg04317399	0.23	0.06	0.24	0.08	0.23	0.10	**0.125**	
HOXD3	cg00005847	0.22	0.04	0.22	0.05	0.22	0.07	**0.116**	
HOXC6	cg10593493	0.05	0.01	0.04	0.00	0.05	0.01	**0.104**	
HOXB4	cg04609859	0.11	0.07	0.11	0.06	0.12	0.08	**0.100**	
HOXD4	cg18750960	0.23	0.07	0.20	0.10	0.22	0.14	**0.091**	
HOXA13	cg06397837	0.07	0.05	0.08	0.03	0.07	0.02	**0.086**	
HOXD12	cg23130254	0.31	0.08	0.27	0.08	0.30	0.14	**0.085**	
HOXB7	cg06493080	0.10	0.05	0.09	0.02	0.09	0.02	**0.079**	
HOXC5	cg00187380	0.06	0.01	0.05	0.01	0.06	0.03	**0.076**	
MSX1	cg11930592	0.08	0.04	0.09	0.06	0.07	0.08	**0.073**	
HOXD13	cg19325985	0.51	0.06	0.48	0.06	0.52	0.08	**0.073**	
HOXA2	cg26069745	0.25	0.07	0.17	0.11	0.24	0.12	**0.068**	
HOXB4	cg08089301	0.06	0.04	0.07	0.02	0.06	0.01	**0.063**	
HOXB4	cg25145670	0.08	0.01	0.09	0.01	0.08	0.01	**0.058**	
HOXA3	cg25426743	0.56	0.06	0.58	0.07	0.57	0.11	**0.052**	
HOXC8	cg05022306	0.06	0.01	0.05	0.01	0.06	0.01	**0.037**	
HOXD8	cg21815667	0.31	0.11	0.23	0.08	0.30	0.15	**0.037**	
HOXB7	cg09357097	0.11	0.05	0.11	0.07	0.11	0.05	**0.026**	
MSX1	cg20891301	0.73	0.03	0.77	0.04	0.73	0.05	**0.017**	
HOXD1	cg19001226	0.10	0.03	0.10	0.02	0.10	0.03	**0.013**	
HLX1	cg13170032	0.20	0.03	0.21	0.03	0.20	0.03	**0.013**	
MSX1	cg20588069	0.57	0.06	0.57	0.05	0.57	0.11	**0.013**	
HOXB4	cg06760035	0.06	0.02	0.05	0.01	0.06	0.03	**0.005**	
